# H3K9 tri-methylation at *Nanog* times differentiation commitment and enables the acquisition of primitive endoderm fate

**DOI:** 10.1242/dev.201074

**Published:** 2022-09-09

**Authors:** Agnès Dubois, Loris Vincenti, Almira Chervova, Maxim V. C. Greenberg, Sandrine Vandormael-Pournin, Déborah Bourc'his, Michel Cohen-Tannoudji, Pablo Navarro

**Affiliations:** 1 Department of Developmental and Stem Cell Biology, Institut Pasteur, Université Paris Cité, CNRS UMR3738, Epigenomics, Proliferation, and the Identity of Cells Unit, F-75015 Paris, France; 2 Department of Genetics and Developmental Biology, Institut Curie, PSL Research University, INSERM, CNRS, 75005 Paris, France; 3 Université Paris Cité, CNRS, Institut Jacques Monod, F-75013 Paris, France

**Keywords:** ERK, H3K9me3, Nanog, ZFP57, Heterogeneity, Primitive endoderm, Mouse

## Abstract

Mouse embryonic stem cells have an inherent propensity to explore gene regulatory states associated with either self-renewal or differentiation. This property depends on ERK, which downregulates pluripotency genes such as *Nanog*. Here, we aimed at identifying repressive histone modifications that would mark *Nanog* for inactivation in response to ERK activity. We found that the transcription factor ZFP57, which binds methylated DNA to nucleate heterochromatin, is recruited upstream of *Nanog*, within a region enriched for histone H3 lysine 9 tri-methylation (H3K9me3). Whereas before differentiation H3K9me3 at *Nanog* depends on ERK, in somatic cells it becomes independent of ERK. Moreover, the loss of H3K9me3 at *Nanog*, induced by deleting the region or by knocking out DNA methyltransferases or *Zfp57*, is associated with reduced heterogeneity of NANOG, delayed commitment into differentiation and impaired ability to acquire a primitive endoderm fate. Hence, a network axis centred on DNA methylation, ZFP57 and H3K9me3 links *Nanog* regulation to ERK activity for the timely establishment of new cell identities. We suggest that establishment of irreversible H3K9me3 at specific master regulators allows the acquisition of particular cell fates during differentiation.

## Abstract (precis)

**Summary:** A regulatory axis integrating ERK, ZFP57, DNA and H3K9 methylation underlies the transition of *Nanog* expression from heterogeneous and dynamic to irreversibly silenced, enabling differentiation commitment and primitive endoderm specification.

## INTRODUCTION

Mouse embryonic stem cells (ESCs) are derived from pre-implantation embryos and recapitulate numerous properties of the pluripotent inner cell mass of the blastocyst (Martello and Smith, 2014). *In vivo*, the culmination of pluripotency – the ability to give rise to all three somatic germ layers – takes place when the primitive endoderm – a source of extra-embryonic tissues – segregates from the epiblast, the founder of the embryo proper (Chazaud and Yamanaka, 2016). This segregation is strictly controlled by the transcription factor *Nanog*, which is required to form the epiblast (Mitsui et al., 2003; Silva et al., 2009) and, additionally, stimulates FGF4 production. This extracellular signal is then transduced into neighbouring cells by ERK (MAPK1) activity, silencing *Nanog* and opening a window of opportunity to undergo commitment into primitive endoderm differentiation (Chazaud et al., 2006; Nichols et al., 2009; Yamanaka et al., 2010; Frankenberg et al., 2011; Saiz et al., 2016; Bessonnard et al., 2017). This binary cell-fate decision is characterised by substantial heterogeneity of NANOG expression, which creates the conditions required for epiblast and primitive endoderm specification (Chazaud et al., 2006). Subsequently, *Nanog* expression is downregulated in the epiblast, eliciting the establishment of somatic differentiation (Chambers et al., 2003). *In vitro*, ESCs also exhibit extensive *Nanog* heterogeneity, characterised by a subpopulation expressing no or extremely low levels of NANOG. Whereas NANOG-positive cells self-renew efficiently, NANOG-negative cells exhibit a propensity to differentiate even though they remain uncommitted and can spontaneously revert to the *Nanog*-expressing state (Chambers et al., 2007; Singh et al., 2007; Kalmar et al., 2009; Canham et al., 2010; Abranches et al., 2014). Notably, NANOG-negative cells spontaneously generated in culture or by homozygous targeted deletion show increased differentiation propensity towards both primitive endoderm and somatic fates (Chambers et al., 2007; Singh et al., 2007; Kalmar et al., 2009; Canham et al., 2010; Abranches et al., 2014).

NANOG heterogeneity has been proposed to result from two mechanisms: (1) from the architecture and the topology of the pluripotency network (Navarro et al., 2012; Karwacki-Neisius et al., 2013), and (2) from extrinsic cues, such as LIF/STAT3, WNT/GSK3b and FGF/ERK signalling (Kalmar et al., 2009; Wray et al., 2011; Marks et al., 2012; Abranches et al., 2014). For both, specific regulatory properties and their inherent stochastic nature have been suggested to play a role (Martinez Arias and Brickman, 2011). Nevertheless, ERK activity has emerged as a key trigger of *Nanog* heterogeneity, in line with its general role in eliciting exit from pluripotency (Kunath et al., 2007; Schröter et al., 2015). However, little is known about the chromatin-based aspects of *Nanog* heterogeneity. More specifically, it is unknown whether specific chromatin modifications (Jenuwein and Allis, 2001) contribute to stabilisation of the NANOG-negative state, which has been shown to be perpetuated for several cell divisions during self-renewal. Indeed, temporal analysis of NANOG fluctuations across cell generations has shown that the progeny of NANOG-negative cells is enriched in cells that maintain *Nanog* silencing, even though they can revert back and re-express NANOG (Hastreiter et al., 2018). This stability of the NANOG-negative state sets *Nanog* heterogeneity apart from other phenomena of gene expression heterogeneity, generally characterised by fast-switching dynamics resulting from intrinsic and extrinsic noise or encoded in regulatory networks themselves (Huang, 2009; Balazsi et al., 2011).

In this study, we aimed at identifying histone modifications associated with gene repression that would be: (1) differentially enriched at the *Nanog* locus when it is active or inactive; (2) controlled by the signalling pathways associated with heterogeneity; and (3) heritable from mother to daughter cells (Gonzalez et al., 2021). We found histone H3 lysine 9 tri-methylation (H3K9me3; Jambhekar et al., 2019) enriched between the *Nanog* promoter and its −5 kb enhancer (Loh et al., 2006; Levasseur et al., 2008) to fulfil these criteria. We also found that H3K9me3 at NANOG depends on DNA methylation and on the binding of ZFP57, a transcription factor known to nucleate heterochromatin in ESCs (Quenneville et al., 2011; Zuo et al., 2012; Anvar et al., 2016; Riso et al., 2016; Li et al., 2008). Analysis of independent ESC mutants lacking H3K9me3 at *Nanog* revealed its role in promoting NANOG heterogeneity, commitment into differentiation and, most notably, effective differentiation along the primitive endoderm lineage. Moreover, our data also suggest that during differentiation H3K9me3 at *Nanog* becomes independent of ERK activity. Hence, we propose that the timely establishment of ERK-independent H3K9me3 at *Nanog* marks commitment into differentiation and impacts cell-fate acquisition in a lineage-dependent manner.

## RESULTS

### ERK-dependent, mitotically stable, H3K9me3 at the *Nanog* locus in ESCs

**Fig. 1.**
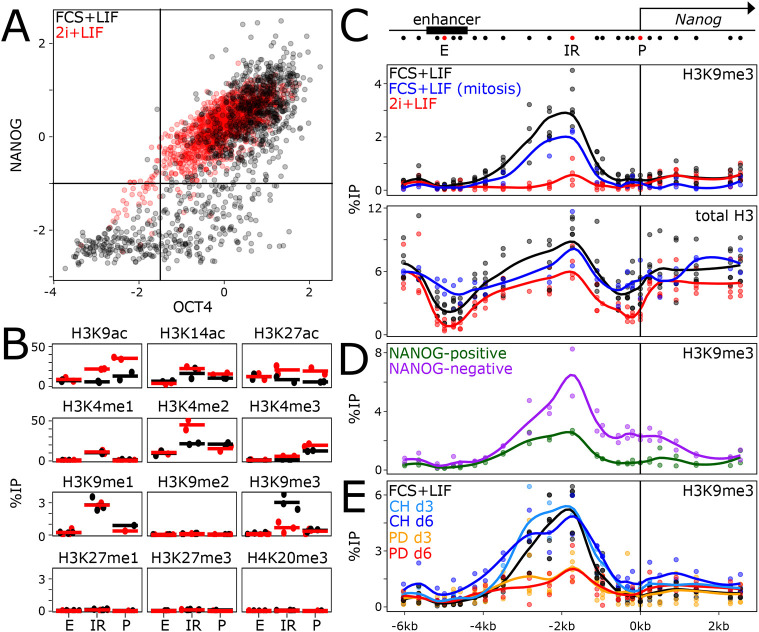
**Mitotically stable, ERK-dependent H3K9me3 at the *Nanog* locus.** (A) Quantification of OCT4 (*z*-score; *x*-axis) and NANOG (*z*-score; *y*-axis) levels as assessed by immunostaining of ESCs cultured in FCS+LIF (black, *n*=1125) and 2i+LIF (red, *n*=1445). The difference in NANOG levels distribution was assessed with Kolmogorov–Smirnov** **test (*P*<2.2e−16). (B) ChIP-qPCR of several histone modifications, as indicated, at three positions of the *Nanog* locus (the *Nanog* promoter, P; the *Nanog* −5 kb enhancer, E; an intervening region, IR, located 1.7 kb of the *Nanog* transcription start site; see the red dots on the schematic in C) in ESCs cultured in FCS+LIF (black) or in 2i+LIF (red). Each dot represents the percentage of immunoprecipitation (%IP; *y*-axis) for independent replicates and the bar the corresponding mean. The loss of H3K9me3 observed at IR in 2i+LIF was assessed with unpaired one-tailed Student's *t*-test (*P*=0.006135). (C-E) Extended profile of H3K9me3 or total H3, as indicated, across the *Nanog* locus (*x*-axis represents genomic distances in kb with respect to the *Nanog* transcription start site, as schematised above), in ESCs cultured as indicated in each plot. Each dot represents the percentage of immunoprecipitation (%IP; *y*-axis) for independent replicates and the line the corresponding Loess regression. The differences in H3K9me3 enrichments at the IR region (from −3.5 kb to −0.9 kb) were analysed with unpaired one-tailed Student's *t*-test combining all individual primers (*P*=2.316e-09 for FCS+LIF versus 2i+LIF; *P*=0.001528 for NANOG negative versus positive; *P*=1.367e−06 for untreated versus PD_d3). The increase in H3K9me3 at the promoter region between NANOG-positive versus -negative cells was analysed as for the IR but using primers within the −0.5 kb to 0 kb region (*P*=2.447e−07). d, day.

To explore the involvement of chromatin marks potentially distinguishing active and inactive *Nanog* states, we first performed a comparison of ESC populations exhibiting heterogeneity, cultured in the presence of fetal calf serum (FCS) and leukaemia inhibitory factor (LIF) (FCS+LIF) (Fig. 1A) and highly homogeneous populations obtained by double inhibition of ERK and GSK3b (2i+LIF; Fig. 1A). Although several euchromatic marks associated with active transcriptional states were found to be more enriched in 2i+LIF, as expected, we observed a single repressive mark, H3K9me3, to be enriched in FCS+LIF and lost in 2i+LIF (Fig. 1B). H3K9me3 was present at neither the promoter (P in Fig. 1B) nor the enhancer (E in Fig. 1B); rather, we detected H3K9me3 in the intervening region (IR in Fig. 1B). To characterise H3K9me3 further, we performed a high-resolution analysis of the *Nanog* locus (Fig. 1C, top), which confirmed the presence of a robust peak of H3K9me3 between the enhancer and the promoter in the FCS+LIF condition exclusively (Fig. 1C). Analysis of total H3 confirmed the specificity of H3K9me3 enrichment (Fig. 1C), which cannot be merely attributed to changes in nucleosome positioning or occupancy. Moreover, we observed good retention of H3K9me3 at *Nanog* in mitotic ESCs (Fig. 1C), in which drastic changes in nucleosomal densities could also be observed at the *Nanog* enhancer (Fig. 1C; Festuccia et al., 2019). Next, we used a previously described *Nanog*-GFP reporter (Chambers et al., 2007) to sort *Nanog*-positive and -negative ESCs by fluorescence-activated cell sorting (FACS). We observed that H3K9me3 was more prominent in *Nanog*-negative cells, with clear spreading towards the promoter (Fig. 1D). H3K9me3 was also found present, albeit at low levels, in *Nanog*-positive cells, obtained either by FACS (Fig. 1D) or by taking advantage of a puromycin selection cassette linked to the *Nanog*-GFP allele (Fig. S1A,B). Finally, we assessed the temporal ERK and GSK3b dependencies of H3K9me3 at *Nanog*. After 3 days of ERK inhibition with PD0325901, which induces high and homogeneous NANOG expression (Fig. S1C,D), H3K9me3 was significantly reduced (PD in Fig. 1E), whereas even after 6 days of GSK3b inhibition with CHIR99021, H3K9me3 levels remained globally unchanged (CH in Fig. 1E). Hence, the repressive H3K9me3 mark exhibits properties that indicate it may play a role in *Nanog* heterogeneity as it is readily dependent on ERK activity, lost in homogeneous NANOG populations, over-enriched in *Nanog*-negative cells, and maintained during mitosis.

### H3K9me3 at *Nanog* impacts NANOG heterogeneity

**Fig. 2.**
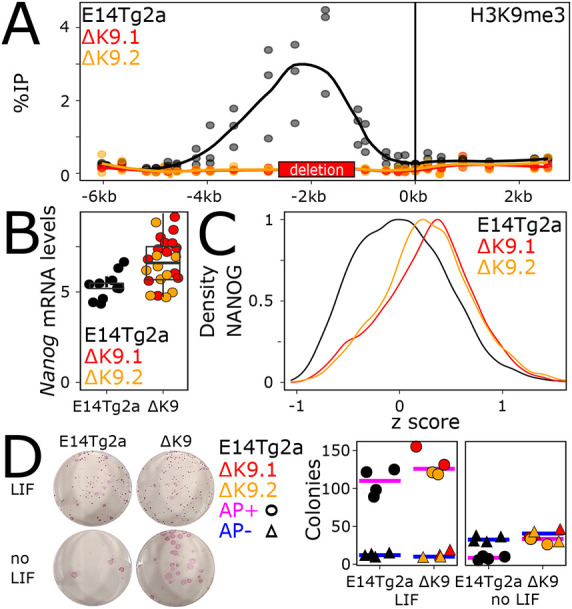
**The *Nanog* region harbouring H3K9me3 enables heterogenous NANOG expression.** (A) H3K9me3 profile across the *Nanog* locus, presented as in Fig. 1C, in wild-type ESCs (E14Tg2a, black) and in two mutant derivatives (ΔK9.1 and ΔK9.2, in red and orange, respectively), carrying a deletion of the region enriched for H3K9me3 (red box on the *x*-axis). The differences in H3K9me3 enrichments at the non-deleted parts of the IR region were analysed with unpaired one-tailed Student's *t*-test combining all individual primers and the two mutant clones (*P*=0.001036). (B) Expression of *Nanog* mRNA levels (normalised to *Tbp*) assessed by RT-qPCR in wild-type (E14Tg2a, black) and mutant (red and orange) clones. Each dot represents the level of *Nanog* mRNA for independent replicates and the boxplots the corresponding median (bar), 25-75% percentiles (box) and 1.5× the inter-quartile range (whiskers). The difference between E14Tg2a and ΔK9 cells was assessed with unpaired one-tailed Student's *t*-test (*P*=0.0002491). (C) Histogram representing the density (*y*-axis) of NANOG expression levels [*x*-axis; log2(mean_intensity) corrected to the E14Tg2a median of each experiment, *n*=2] in wild-type (E14Tg2a, black; *n*=8191) or mutant (ΔK9.1 and ΔK9.2 in red and orange, respectively; *n*=3835, 3812) cells as assessed by immunostaining. The difference in NANOG distributions was analysed with a Kolmogorov–Smirnov** **test (*P*<2.2e−16 for each clone versus E14Tg2a). (D) Representative alkaline phosphatase staining of ESC colonies for the indicated cell lines and culture conditions. The plot shows the number of alkaline phosphatase (AP)-positive (circles) and -negative colonies (triangles) in wild-type (E14Tg2a, black) and ΔK9 (red and orange) cells. Each dot represents an independent replicate and the bar the corresponding median for AP-positive (pink) or AP-negative (blue) colonies. The small difference in the number of AP-positive colonies between E14Tg2a and mutant cells cultured in FCS+LIF was not statistically significant (Mann–Whitney test, *P*=0.1714), whereas it was significant in the absence of LIF (*P*=0.01429).

To study the relevance of H3K9me3 at *Nanog*, we used a Crispr/Cas9 approach deleting ∼1.8 kb between the enhancer and promoter (Fig. 2A, red box). Two clones (ΔK9.1 and ΔK9.2) were confirmed as homozygous deletions with a complete absence of H3K9me3 (Fig. 2A). *Nanog* mRNA levels were slightly upregulated in ΔK9 cells (Fig. 2B), which presented a clear shift in NANOG expression, leading to a strong reduction of the proportion of cells expressing no or low NANOG (Fig. 2C), as confirmed at the mRNA level by single-molecule fluorescence in situ hybridisation (smFISH) (Fig. S1E,F). Nevertheless, the loss of heterogeneity was not as prominent as that achieved by ERK inhibition (Fig. S1D), indicating that ERK also inhibits *Nanog* transcription by other means. In line with this, *Nanog* expression further increased upon ERK inhibition in ΔK9 clones (Fig. S1C). Moreover, whereas strong ectopic induction of NANOG leads to improved self-renewal (Chambers et al., 2007), the small upregulation of *Nanog* in ΔK9 cells was associated with a marginal increase in self-renewal efficiency, as determined by clonal assays (Fig. 2D). However, ΔK9 cells were more recalcitrant to efficient differentiation upon LIF withdrawal (Fig. 2D). Next, we performed RNA-sequencing (RNA-seq) analysis to compare wild-type and ΔK9 cells (Table S1), which confirmed a small increase in Nanog expression [false discovery rate (FDR)<0.05; Fig. S2A]. We identified 235 and 402 genes that were up- or downregulated, respectively, in both ΔK9 clones {FDR<0.05 and abs[log2fold change (FC)]>0.3; Fig. S2B}. Although differentially expressed genes exhibited small fold changes (Fig. S2C), consistent with the small increase in *Nanog* expression observed in ΔK9 cells, they were nonetheless found enriched in the vicinity of NANOG-binding regions, compared with regions bound by OCT4 (POU5F1) and SOX2 but not NANOG (Festuccia et al., 2019; Heurtier et al., 2019; Fig. S2D). We conclude that deletion of the region harbouring H3K9me3 at the *Nanog* locus reduces the capacity of ESCs to explore the NANOG-negative state efficiently, leading to minimal gene expression changes and a measurable resistance to differentiation.

### H3K9me3 at *Nanog* controls the timing of commitment into differentiation

**Fig. 3.**
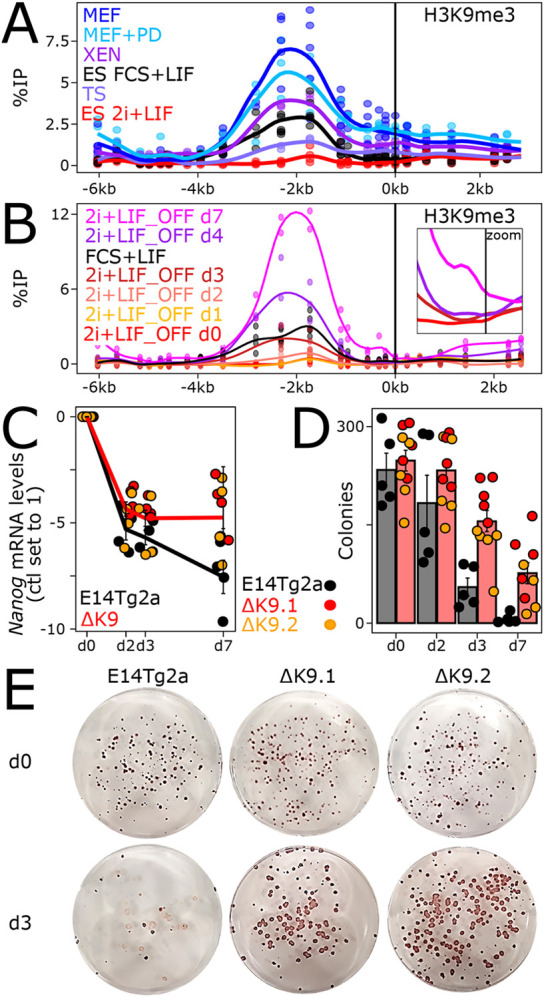
**H3K9me3 at *Nanog* times commitment into differentiation.** (A) H3K9me3 profile across the *Nanog* locus, presented as in Fig. 1C, in the indicated cell lines and conditions. The enrichment of H3K9me3 at either the IR (from −3.5 kb to −0.9 kb) or the promoter region (from −0.5 kb to 0 kb) in non-pluripotent cells was compared with 2i+LIF ESCs with unpaired one-tailed Student's *t*-test combining all individual primers falling in each subregion (*P*=0.002566 and 2.307e−06 for IR and promoter, respectively, for TSCs; *P*=0.001053 and 4.716e−06 for XEN cells; *P*=4.878e−08 and 3.79e−09 for MEFs). (B) Identical profiles for ESCs undergoing differentiation (labelled 2i+LIF_OFF) for the indicated number of days. The same statistical analyses as in A showed that the enrichment at the IR and promoter region was statistically significant from day 3 onwards (*P*=0.00226, 0.0001353, 0.0004633 for the IR, and *P*=0.04536, 0.0004678, 0.002106 for the promoter, at days 3, 4 and 7, respectively). The inset focuses on the promoter region (−1 kb to 0.5 kb) to better appreciate H3K9me3 spreading from day 0 to day 3, 4 and 7. (C) *Nanog* log2 relative mRNA levels (day 0 set to 1) measured by RT-qPCR and normalised to *Tbp*, during ESC differentiation in the indicated cell lines. Each dot represents an independent replicate and the line the corresponding mean with s.e.m. The comparative analysis of the temporal reduction of *Nanog* between E14Tg2a and ΔK9 clones (unpaired one-tailed Student's *t*-test) showed that it was statistically significant only at day 7 (*P*=0.08368, 0.05471 and 0.01324 at days 2, 3 and 7, respectively). Moreover, the reduction observed for E14Tg2a between days 3 and 7 was also found to be significant (*P*=0.03026). (D) Number of alkaline phosphatase-positive colonies obtained after switching to 2i+LIF for wild-type (E14Tg2a, black) and ΔK9 (red and orange) cells seeded clonally and differentiated for the number of days indicated on the *x*-axis. Each dot represents an independent replicate and the histogram the corresponding mean and s.e.m. Differences in clonogenicity were assessed with a Mann–Whitney test (*P*=0.2198, 0.001332, 0.002531 for days 2, 3 and 7, respectively). (E) Representative alkaline phosphatase staining of ESC colonies cultured in 2i+LIF after 0 (top) or 3 (bottom) days of differentiation for the indicated cell lines. d, day.

We next aimed at characterising the status of H3K9me3 at *Nanog* in non-pluripotent cells. First, we established the H3K9me3 profiles over the *Nanog* locus in several cell types in which *Nanog* has been silenced during development (Fig. 3A). H3K9me3 was systematically found to be enriched between the *Nanog* enhancer and promoter, albeit at different levels. In trophectoderm stem cells (TSCs), the levels found at *Nanog* were lower than in FCS+LIF ESCs, except at the *Nanog* promoter where some spreading was detected. In extra-embryonic endoderm (XEN) cells, the levels of H3K9me3 were higher, exhibiting spreading towards the promoter. Finally, in mouse embryonic fibroblasts (MEFs), the level of H3K9me3 was particularly high, with prominent invasion of the *Nanog* promoter and gene body. Therefore, we conclude that, although H3K9me3 is found at *Nanog* in the three cell types analysed, its absolute levels and the degree of spreading towards the promoter are variable. This suggests that a certain level of developmental specificity impacts H3K9 methylation at *Nanog*. Moreover, and in contrast to ESCs, inhibition of ERK in MEFs did not abolish H3K9me3 at *Nanog*, which remained robustly enriched (Fig. 3A). This indicates that during differentiation, when *Nanog* silencing becomes irreversible, H3K9me3 at *Nanog* is liberated from its strict dependency on ERK.

We then aimed at assessing the dynamics of H3K9me3 at *Nanog* during ESC differentiation. We used a simple protocol starting from 2i+LIF (absence of H3K9me3) and based on the withdrawal of LIF and ERK/GSK3b inhibitors (Fig. S3A,B). We observed a step-wise increase of H3K9me3 (Fig. 3B): if it remained low during the first 48 h, it suddenly appeared after 3 days and increased at days 4 and 7, when low but clear signs of spreading to the promoter were also observed. In ΔK9 clones, however, H3K9me3 remained absent during differentiation (Fig. S3C). Somehow, unexpectedly, the appearance of H3K9me3 at day 3 did not correlate with a particularly strong reduction of *Nanog* expression (Fig. 3C). In fact, we observed *Nanog* downregulation taking place largely during the first 48 h, in the absence of high levels of H3K9me3. However, although *Nanog* expression continued to decrease during differentiation of wild-type cells, when H3K9me3 further increased and then spread to the *Nanog* promoter (Fig. 3B) ΔK9 cells displayed a stabilisation of low *Nanog* expression after the sharp decrease occurring during the first 2 days (Fig. 3C), despite efficient differentiation (Fig. S3A,B). Immunofluorescence analyses further indicated that the retention of low but measurable NANOG expression affected the vast majority of ΔK9 cells (Fig. S4). This different global behaviour of *Nanog* expression in ΔK9 clones, temporally correlated with the time at which H3K9me3 is first established and subsequently spreads to the promoter in wild-type cells, prompted us to determine whether commitment into differentiation – the moment at which cells cannot easily come back to an undifferentiated state – was altered in ΔK9 cells. For this, we seeded wild-type and ΔK9 cells at clonal density and after 2, 3 or 7 days of differentiation, we replaced the culture medium with 2i+LIF: only cells that have not yet irreversibly lost their capacity to self-renew will survive, proliferate and form undifferentiated, alkaline phosphatase-positive colonies (Kalkan et al., 2017; Fig. 3D,E). In wild-type cells, we observed a striking coincidence of the time of commitment, taking place between days 2 and 3, with the appearance of H3K9me3 at *Nanog*. In ΔK9 clones, however, we observed a significant number of alkaline phosphatase-positive colonies after 3 and even 7 days of differentiation, indicating a delay in commitment. Altogether, these analyses suggest that in ESCs cultured in 2i+LIF, H3K9me3 at *Nanog* is established during differentiation, when it marks the irreversible commitment into effective differentiation. However, it is not strictly required for differentiation per se and rather enables the appropriate timing of commitment.

### ΔK9 cells exhibit delayed differentiation

**Fig. 4.**
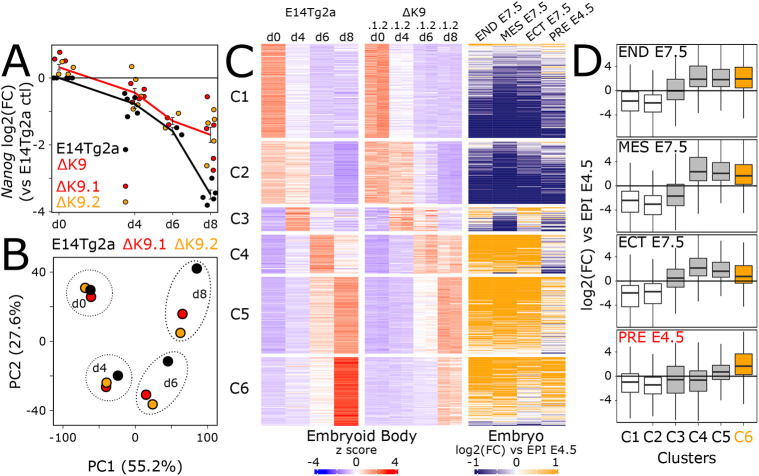
**The lack of H3K9me3 at *Nanog* leads to delayed differentiation.** (A) Log2 *Nanog* mRNA levels measured by RT-qPCR and normalised to *Tbp* during EB differentiation in the indicated cell lines. Each dot represents an independent replicate and the line the corresponding mean with s.e.m. Expression differences between E14Tg2a and ΔK9 clones were evaluated at each day of differentiation with unpaired one-tailed Student's *t*-tests (*P*=0.002143, 0.0485, 0.06709, 7.367e−06 for days 0, 4, 6 and 8, respectively). (B) PCA of 16,336 transcripts quantified by RNA-seq (TPM >1 in at least one sample) in wild-type (E14Tg2a, black; *n*=3) and ΔK9 (red and orange; *n*=3 for each) cells undergoing EB differentiation for the indicated days. (C) Heatmap of 4100 transcripts displaying gene expression changes during EB differentiation [FDR<0.05 and abs(log2FC)>1 in at least one comparison to undifferentiated cells; *n*=3 for each cell line], clustered by k-means after *z*-score normalisation. Left: Relative gene expression (*z-*score) in wild-type (E14Tg2a) and ΔK9 cells during EB differentiation. Right: Corresponding log2FC of each developmental stage indicated at the top versus E4.5 epiblast, as previously reported (Argelaguet et al., 2019). (D) Boxplot (median; 25-75% percentiles; 1.5× the inter-quartile range) of the log2FC shown in C for each cluster. Cluster C6 is highlighted in orange. The highest distribution of log2FC for PRE E4.5 for C6 with respect to the other clusters was assessed with a Kolmogorov–Smirnov** **test (*P*<1e−07 for all comparisons). d, days; ECT, ectoderm; END, endoderm; EPI: epiblast; MES, mesoderm; PRE, primitive endoderm.

Given the delay in differentiation commitment observed in ΔK9 clones, we monitored the expression of several genes reflecting the loss of pluripotency and the transition to a differentiated state (Kalkan et al., 2017; Fig. S3B). Whereas naïve pluripotency genes [*Esrrb*, *Klf4*, *Prdm14*, *Rex1* (*Zfp42*)] showed a less drastic downregulation, mimicking *Nanog* expression, differentiation markers (*Fgf5*, *Dnmt3b*, *Otx2*, *Wnt3*) showed slightly delayed dynamics. Next, we differentiated wild-type and ΔK9 cells into embryoid bodies (EBs), a paradigm that recapitulates the establishment of multiple lineages. At the morphological level, we observed ΔK9 EBs to be often characterised by defective sealing at their periphery (Fig. S5A, top). Moreover, cellular outgrowths derived from ΔK9 EBs also exhibited less morphological typologies compared with those derived from wild-type EBs, suggesting altered multi-lineage differentiation (Fig. S5A, bottom). Gene expression analyses of EBs after days 4, 6 and 8 of differentiation confirmed the attenuated downregulation of *Nanog* expression (Fig. 4A). Moreover, principal component analysis (PCA) analysis of RNA-seq profiling (Table S1) highlighted a transcriptome-wide delay of both ΔK9 clones, starting at day 4 and progressively increasing through time (Fig. 4B). Gene ontology analysis of the top 1000 loadings of the PCA identified focal adhesion genes among the most enriched cellular components (GO:0005925; *P*<10^−5^), in line with our morphological observations (Fig. S5A). Therefore, the lack of H3K9me3 at *Nanog* is strongly associated with delayed differentiation, as evaluated with three distinct differentiation paradigms (Fig. 2D, Fig. 4B, Fig. S3B).

### The absence of H3K9me3 at *Nanog* leads to major defects in primitive endoderm differentiation

To explore further the molecular nature of the defects observed during EB differentiation, we first identified around 4000 genes displaying temporal gene expression changes in either differentiating wild-type or mutant cells compared with their undifferentiated controls [FDR<0.05, abs(log2FC)>1; Table S1]. These genes were then clustered using k-means; this allowed us to identify six groups of genes displaying different expression dynamics during EB differentiation (Fig. 4C, left; Table S1). Although all identified clusters underscored the delay into differentiation, one in particular, cluster C6, showed a blatant attenuation of gene upregulation at day 8. To characterise each cluster relative to known developmental trajectories, we plotted the fold change reported in a previous study (Argelaguet et al., 2019), in which embryonic endoderm, mesoderm and ectoderm cells of embryonic day (E) 7.5 embryos, as well as primitive endoderm cells of E4.5 embryos, were directly compared with the E4.5 epiblast (Fig. 4C, right). Whereas clusters C1 and C2 showed a marked downregulation upon epiblast differentiation into all lineages, clusters C3 to C6 displayed a clear upregulation in at least one E7.5 lineage. Notably, cluster C6, which shows the strongest ΔK9 versus wild-type differences, was the only one enriched for genes displaying a prominent upregulation in the primitive endoderm with respect to the epiblast (Fig. 4D). Analysis of individual primitive endoderm markers during EB differentiation confirmed the altered induction of these genes (Fig. S5B,C).

**Fig. 5.**
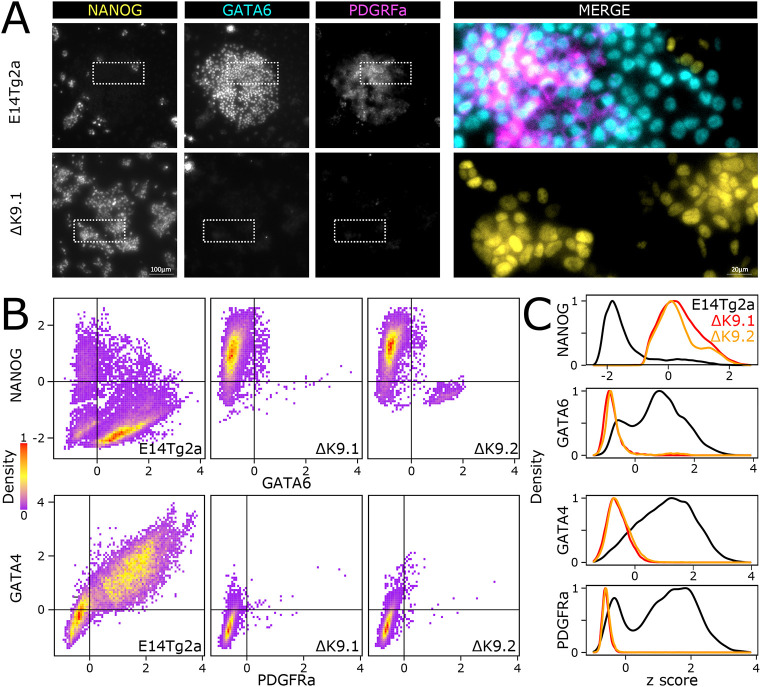
**Lack of H3K9me3 at *Nanog* abolishes primitive endoderm differentiation.** (A) Representative immunostaining of ESCs (E14Tg2a, top; ΔK9.1, bottom) subject to a directed differentiation protocol into primitive endoderm. Boxed areas are shown at higher magnification, with signals merged, on the right. (B,C) Quantification of dual immunostaining for NANOG and GATA6 or GATA4 and PDGRFα in E14Tg2a (*n*=19,507, 20,641), ΔK9.1 (*n*=11,348, 19,476) and ΔK9.2 (*n*=9713, 19,742) cells differentiated as in A. *P*<2.2e−16 (Kolmogorov–Smirnov** **tests) for all E14Tg2a versus ΔK9 comparisons.

Finally, in light of these results, we wanted to ascertain whether the defective primitive endoderm signature identified in EBs implies a deficiency in the capacity of ΔK9 clones to engage in primitive endoderm differentiation. Thus, we challenged wild-type and ΔK9 ESCs with a primitive endoderm differentiation protocol (Anderson et al., 2017; Fig. S6). In wild-type cells, but not in ΔK9 clones, we observed the appearance of endoderm-like cell clusters from day 4 onwards. Moreover, ΔK9 clones showed increased cell death. Immunofluorescence of NANOG, GATA6, GATA4 and PDGFRα confirmed NANOG silencing in cells expressing primitive endoderm markers, as expected (Fig. 5). In ΔK9 clones, however, NANOG expression was prominent with only the occasional appearance of cells expressing primitive endoderm markers. Therefore, ΔK9 cells are not able to efficiently undergo directed differentiation into primitive endoderm. We conclude that H3K9me3 is required to stably silence *Nanog* during differentiation and that failing to do so has different consequences depending on the differentiation lineage, with primitive endoderm being particularly sensitive.

### DNA methylation and ZFP57 binding trigger H3K9me3 at *Nanog*

**Fig. 6.**
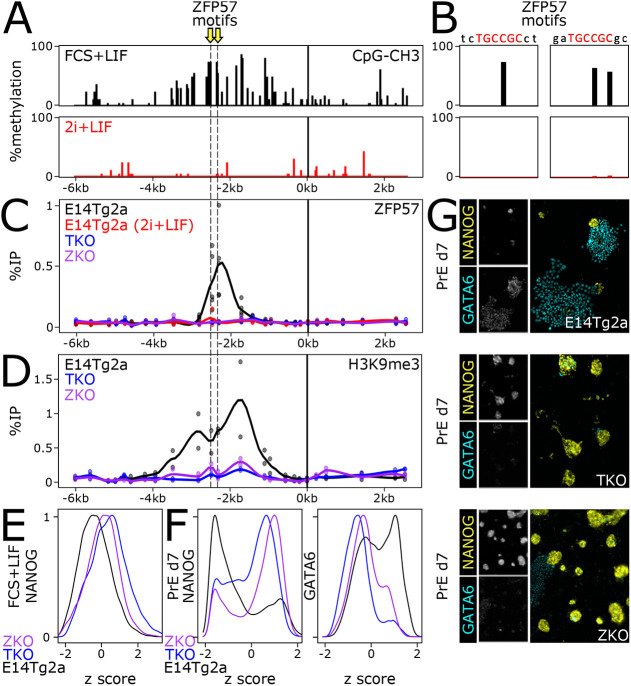
**DNA methylation and ZFP57 binding drive H3K9me3 enrichment at *Nanog*.** (A) Analysis of DNA methylation at the *Nanog* locus, using available datasets in ESCs cultured in FCS+LIF or in 2i+LIF. The position of two ZFP57 motifs is indicated by yellow arrows. (B) Identical analysis but focusing at the two ZFP57 motifs. (C) Analysis of ZFP57 binding by ChIP-qPCR, presented as in Fig. 1C, in the indicated cell lines: E14Tg2a cultured in FCS+LIF (black) or in 2i+LIF (red), triple-negative DNMT knockout ESCs (TKO, blue) and *Zfp57* knockout ESCs (ZKO, purple). (D) Analysis of H3K9me3 at *Nanog* in E14Tg2a, TKO and ZKO ESCs cultured in FCS+LIF, presented as in C. (E) Analysis of NANOG expression in E14Tg2a, TKO and ZKO ESCs. The differential distribution in TKO and ZKO versus E14Tg2a was assessed with Kolmogorov–Smirnov** **tests (*P*<2.2e−16). (F) Analysis of NANOG and GATA6 expression after 7 days of directed differentiation of E14Tg2a, TKO and ZKO into primitive endoderm. Differences in distribution levels were assessed with Kolmogorov–Smirnov tests (*P*<2.2e−16 for both TKO and ZKO versus E14Tg2a). (G) Representative immunostaining of E14Tg2a, TKO and ZKO subjected to a directed differentiation protocol into primitive endoderm. Insets show individual signals, and main panels merged signals.

To investigate the mechanisms by which H3K9me3 is established at the *Nanog* locus, we used the Cistrome database (Zheng et al., 2019) to identify candidate factors binding the region displaying maximal levels of H3K9me3 at *Nanog*. Among the identified factors (Fig. S7A), ZFP57, KAP1 (TRIM28) and DNMT3a appeared particularly relevant. Indeed, ZFP57 has been shown to bind its cognate DNA motif (TGCCGC) only when the CpG is methylated (underlined) and then recruit KAP1, DNA methylases and H3K9 methylases to nucleate heterochromatin formation (Quenneville et al., 2011; Zuo et al., 2012; Anvar et al., 2016; Riso et al., 2016; Li et al., 2008). Two perfect ZFP57 motifs were identified at the exact region showing H3K9me3 in ESCs (Fig. 6A). Moreover, analysis of DNA methylation datasets (Domcke et al., 2015) showed that the two CpGs required for ZFP57 binding are methylated in FCS+LIF but not in 2i+LIF (Fig. 6A,B), as expected given the reduced expression of DNMTs upon ERK inhibition (Li et al., 2016; Spindel et al., 2021 preprint) and the global loss of DNA methylation in 2i+LIF-cultured ESCs (Leitch et al., 2013; Grabole et al., 2013). Hence, we aimed at profiling ZFP57 binding by chromatin immunoprecipitation-quantitative PCR (ChIP-qPCR). We observed robust recruitment of ZFP57 at the H3K9me3-enriched region in ESCs cultured in FCS+LIF but not in 2i+LIF (Fig. 6C,D). Using previously described *Zfp57* knockout ESCs (ZKO; Riso et al., 2016) we were able to confirm the specificity of our assay (Fig. 6C,D). Moreover, upon knockout of all three DNMT genes [*Dnmt1/3a/3b*; triple knockout (TKO)] and the ensuing loss of DNA methylation (Fig. S7B,C), we also observed a complete loss of ZFP57 binding (Fig. 6C). As expected, in both TKO and ZKO cells H3K9me3 at *Nanog* was completely abrogated (Fig. 6D), establishing that it is triggered by a canonical mechanism dependent on DNA methylation and ZFP57 recruitment. Finally, we aimed to address whether the loss of H3K9me3 at *Nanog* triggered by either DNMT or *Zfp57* knockouts is accompanied by changes in NANOG heterogeneity and in the capacity of ESCs to differentiate into primitive endoderm derivatives, as shown in our ΔK9 mutant cells. In both knockouts, we observed a clear increase of NANOG expression before differentiation, depleting the NANOG-low compartment (Fig. 6E), and a strong attenuation of both NANOG downregulation and induction of GATA6 upon directed differentiation into primitive endoderm (Fig. 6F,G), reproducing the effects observed in ΔK9 ESCs. This reinforces the idea that H3K9me3 at *Nanog* is required to lock the *Nanog* silent state during differentiation, an event that is particularly important for proper primitive endoderm differentiation.

## DISCUSSION

Gene expression heterogeneity has emerged as a main motor of lineage diversification during development, particularly in stem cell and progenitor populations: upon appropriate stimuli, these expression differences are translated into an effective decision-making process that culminates in commitment (Huang, 2009; Martinez Arias and Brickman, 2011; Balazsi et al., 2011). When these heterogeneities are dynamic, such as in the case of *Nanog* and ESCs, the fluctuating expression is eventually fixed. A likely model accounting for the transition from reversible to irreversible silencing involves epigenetic repression, in the sense of heritable chromatin states incompatible with transcription that do not depend on the original triggers (Berger et al., 2009). The acquisition of epigenetic repression, including H3K9me3 (Nicetto and Zaret, 2019), is indeed perceived as a general means to restrict developmental fate choices as cells differentiate (Yadav et al., 2018). Accordingly, we show that *Nanog* silencing during differentiation is accompanied by H3K9me3, which irreversibly locks its expression and thus contributes to commitment into differentiation. Two observations, however, indicate that additional complexity may characterise both *Nanog* silencing and its developmental implications.

Although epigenetic repression is often instated *de novo* during differentiation, for example at *Oct4* (Feldman et al., 2006), the regulation of *Nanog* appears to involve an intermediary state in which H3K9me3 is already established but not yet fixed. In undifferentiated cells, H3K9me3 is readily detected at *Nanog*, particularly in *Nanog*-negative cells. Moreover, it is transferred from mother to daughter cells during mitosis. However, it is strictly dependent upon ERK signalling, the main driver of NANOG heterogeneity. This dependency is likely mediated by the impact of ERK on DNA methylation (Leitch et al., 2013; Grabole et al., 2013; Li et al., 2016; Spindel et al., 2021 preprint), in this case of two motifs of the ZFP57 transcription factor, which recruits proteins involved in H3K9 methylation (Li et al., 2008; Quenneville et al., 2011; Zuo et al., 2012; Anvar et al., 2016; Riso et al., 2016). By integrating ERK activity with mitotically stable H3K9 methylation, ZFP57 may confer to *Nanog* silencing the required stability to be inherited and, at the same time, sufficient flexibility to revert back to transcriptional activity. During differentiation (at least as judged by the analysis of embryonic fibroblasts), H3K9me3 becomes independent of ERK and, with respect to ERK, irreversible. Therefore, even if subjected to variations in ERK stimuli, *Nanog* will remain silent. Despite these considerations, we suggest that H3K9me3 at *Nanog* becomes epigenetic exclusively during differentiation, when it is no longer dependent on its original trigger. Hence, our data point to an integrated network of ERK signalling, DNA methylation and ZFP57 binding as the mechanism responsible for H3K9me3 enrichment at *Nanog*. This scenario, whereby DNA methylation plays a central role, is compatible with the kinetics of H3K9me3 loss upon ERK inhibition, which are suggestive of passive erasure of CpG methylation. It is also compatible with the incapacity of ESCs to efficiently maintain DNA methylation and H3K9me3 when transiently induced using epigenomic editing tools (Carlini et al., 2022). Whether the transition from reversible to irreversible H3K9me3 is mediated by direct mechanisms operating at the locus or on the H3K9me3-associated machinery, or linked to the general lack of strong epigenetic repression in ESCs (Carlini et al., 2022; Festuccia et al., 2017), remains unknown. Also, it remains possible that the global increase of H3K9me3 taking place during differentiation, which directly depends on the loss of activity of OCT4 and to a lesser extent of NANOG (Bernard et al., 2022), leads to an improvement in the establishment of robust epigenetic repression. Nevertheless, the dependency of H3K9me3 on ERK shown here and previously on OCT4 (Bernard et al., 2022 preprint) is reminiscent of the dependency of other repressive marks, such as H3K27me3, on LIF signalling and NANOG activity (Heurtier et al., 2019), suggesting a general dependance of repressive chromatin marks on more dynamic regulators in undifferentiated ESCs. By displaying regulated dependencies towards signalling and/or transcription factor activity, repressive chromatin modifications may facilitate conditional heritability and excitability (before differentiation commitment) or, by contrast, fixed gene expression states to enable commitment (Festuccia et al., 2017).

*Nanog* is known to counteract differentiation when ectopically expressed at high levels (Chambers et al., 2007). Because the deletion of the region harbouring H3K9me3 leads to a minor increase of NANOG expression, it was not expected to block differentiation. After all, upon the collapse of the pluripotency network triggered by differentiation signals, *Nanog* would lose most of its activators and be downregulated, as we observed. However, the lack of H3K9me3 triggered by the deletion leads to a lack of complete *Nanog* silencing, providing an opportunity to evaluate the importance of fully repressing *Nanog* during differentiation. Similarly, as cells lacking the H3K9me3-enriched region at *Nanog* reduce their heterogeneity in a context in which *Nanog* can nevertheless be downregulated (in contrast to ectopic expression systems), the importance of NANOG heterogeneity in lineage priming can also be inferred from our experimental setup. In this regard, our observation that cells lacking the H3K9me3-enriched region can differentiate rules out a deterministic role for NANOG heterogeneity in the capacity to exit the undifferentiated state. Nevertheless, using multilineage protocols we observed delayed commitment and altered differentiation into all germ layers of cells lacking the H3K9me3-enriched region. Yet the highest consequences affect genes normally upregulated in the primitive endoderm, an observation that was fully confirmed by their incapacity to differentiate efficiently into primitive endoderm using a directed differentiation protocol. Although the existence of other forms of regulation mediated by the deleted region cannot be formally excluded, the fact that DNMT and *Zfp57* knockouts (which have a wild-type *Nanog* locus) phenocopy the loss of NANOG heterogeneity and the alteration of primitive endoderm differentiation suggests that H3K9me3 plays a major role. The differential impact observed for somatic versus primitive endoderm lineages in ESCs further underscores the relative and lineage-specific importance of *Nanog* in repressing differentiation. Furthermore, *Nanog* heterogeneity in ESCs has been proposed to either reflect the heterogeneity observed in early blastocysts, whereby cells of the inner mass can either express NANOG or GATA6, or the early downregulation of *Nanog* taking place around implantation to elicit somatic differentiation events of the epiblast (Chambers et al., 2007; Singh et al., 2007; Kalmar et al., 2009; Canham et al., 2010; Abranches et al., 2014). Indirectly, thus, our results could be interpreted as *Nanog* heterogeneity and its subsequent full silencing being functionally associated with epiblast versus primitive endoderm specification. In this regard, two observations are noteworthy. First, the repressive H3K27me3 mark has been shown to play a preponderant role in downregulating genes that prime ESCs for primitive endoderm differentiation (Illingworth et al., 2016). Second, *Nanog* sustains H3K27me3 in undifferentiated and early differentiating ESCs (Heurtier et al., 2019). In light of these findings and of our observation that the loss of H3K9me3 also alters the differentiation balance between somatic and primitive endoderm lineages, we suggest that a signalling and transcription factor dialogue established through repressive histone methylation contributes to epiblast versus primitive endoderm specification and differentiation. In this model, ERK dynamically controls H3K9me3 at *Nanog*, which sustains H3K27me3 levels and keeps primitive endoderm genes in check, generating reversible and mosaic expression patterns associated with either epiblast or primitive endoderm fates.

Overall, our observations add to the notion that heterochromatin contributes to cell-fate restriction during differentiation processes. They also suggest that these events are more nuanced in their action than anticipated, given the regulation of its dependency on signalling cues in respect to its epigenetic potential and the differential impact that the ensuing stability of the repression may have in different lineages. Whether our findings can be extrapolated to other master regulators of pluripotency or to other developmental transitions represent important new avenues for future research.

## MATERIALS AND METHODS

### Cell culture

ESCs were cultured on gelatine in either FCS+LIF or 2i+LIF and passaged every 3-4 days. All 2i+LIF analyses were performed after at least three passages in 2i+LIF. ESCs were karyotyped and regularly tested *Mycoplasma*-free. MEFs were derived from F1 129sv/129sv E13.5 male embryos and cultured for no more than four passages. XEN cell lines (Artus et al., 2010) were routinely passaged every 4 days. TSC lines (Kunath et al., 2005) were cultured on mitomycined MEFs and passaged every 2-3 days. For N2B27 differentiation (2i_OFF), ESCs were seeded on poly-L-ornithine/laminin-coated, cell-culture-treated surfaces in 2i+LIF medium but omitting LIF and ERK/GSK3b inhibitors. The medium was changed daily. EBs were obtained by seeding cell aggregates onto non-cell-culture-treated dishes. Primitive endoderm differentiation was performed as previously described (Anderson et al., 2017) using activin A, CHIR99021 and LIF. The medium was changed daily. Commitment assays were performed with cells cultured in 2i+LIF and subject to 2i_OFF differentiation; at each differentiation time point, 2i+LIF was added back for seven additional days, after which cells were fixed and stained for alkaline phosphatase activity. Full details are available in supplementary Materials and Methods.

### Generation of ESC knockout lines

To generate ΔK9 clones, we used gRNAs (5′-CAGAGGAGGGCTTAAGAGAT and 5′-CACTCTAACCCAGCTTAAGT) cloned under the control of a U6 promoter in a vector conferring puromycin resistance (Heurtier et al., 2019). These vectors were co-transfected with a Cas9/mCherry expression vector (Addgene plasmid #64324) in E14Tg2a cells, selected with puromycin and FACS-sorted for mCherry fluorescence. Puromycin-resistant and mCherry-positive cells were seeded at clonal density and ∼100 clones were picked 10 days later. Clones were screened by PCR with primers spanning the deletion (Table S2) by real-time qPCR using primers along the *Nanog* locus (Table S2), and by cloning and sequencing of PCR products (Table S2). Two karyotypically normal independent clones, ΔK9.1 and ΔK9.2, were selected for this study. *Dnmt1/3a/3b* TKO cells were generated in E14Tg2a using gRNAs that were previously described (Domcke et al., 2015). The three gRNAs targeting each DNMT gene were cloned in pX459 (Addgene plasmid #48139) and transfected into E14Tg2a cells. After puromycin selection, ∼96 colonies were picked and screened by DNA digestion with the DNA methylation-sensitive restriction enzyme HpaII. DNA methylation mutant clones were further confirmed by Sanger sequencing (Table S2). For the TKO clone used in this study, complete loss of DNA methylation was confirmed by the luminometric methylation assay (LUMA; Karimi et al., 2006), which was performed exactly as described (Walter et al., 2016).

### FACS

Nanog-GFP cells (TNG; Chambers et al., 2007) were sorted using a Moflo Astrios with the ‘highest % of purity’ parameter selected. After sorting, GFP-negative and GFP-positive sorted populations were re-processed, with the same parameters used for sorting, to check the purity of each fraction (>95%).

### Imaging analyses

To enable direct comparison of undifferentiated E14Tg2a with ΔK9, TKO and ZKO cells, cells were individually incubated either with Rhodamine Red (E14Tg2a) or Deep Red (ΔK9 or TKO or ZKO) dyes, collected, mixed at a 1:1 ratio, seeded and cultured for ∼6 h. Cells were then fixed in 4% formaldehyde for 10 min and used for immunostaining. Differentiating cells were processed separately. Antibodies used for all immunostaining experiments are listed in Table S2. Imaging was performed with an inverted Nikon Eclipse X microscope equipped with ×20/0.45 (WD 8.2-6.9) objective, LUMENCOR excitation diodes, Hamamatsu ORCA-Flash 4.0LT camera and NIS Elements 4.3 software. Quantifications were performed using CellProfiler (Carpenter et al., 2006). For smFISH analyses, cells were grown at low density in medium without phenol-Red, collected by trypsinisation, fixed, cytospun and stored in 70% ethanol at 4°C until use. The slides were dehydrated and hybridised for 24 h at 37°C with the *Nanog* mRNA probe (Stellaris Probe Designer version 4.2 on Biosearch Technologies). Image stacks (0.5 μm gap) were acquired using a Nikon Eclipse X microscope equipped with ×63 oil immersion objective (N.A1.4), LUMENCOR excitation diodes, Hamamatsu ORCA-Flash 4.0LT camera and NIS Elements 4.3 software. The analysis was performed using ImageJ and CellProfiler. Additional details are available in supplementary Materials and Methods.

### Gene expression analyses

RNA extraction and DNase treatment were performed with the NucleoSpin RNA Mini kit (MACHEREY-NAGEL) according to the manufacturer's protocol. Reverse transcription was performed with 1 µg of total RNAs with random hexamers following manufacturer's protocol (Roche). Real-time quantitative PCR was performed in a LightCycler 480 (Roche) using LightCycler 480 SYBR Green I Master (Roche) and normalised to *Tbp* (see Table S2 for primer sequences). Stranded, poly-A-selected RNA-seq libraries were prepared and sequenced (paired-end 150 bp reads; around 50 million each) by Novogene UK. Reads were aligned to the mm10 genome and transcripts per million (TPM) computed. All differential expression tests were run with DESeq2 (Love et al., 2004). For EB differentiation, we considered genes with absolute log2FC>1 and FDR<0.05 at any day of the differentiation versus undifferentiated cells for either E14Tg2a or ΔK9 cells. k-means clustering was computed with R using the function k-means with options *k*=6, nstart=50, iter.max=50. Only differentially expressed genes as identified during EB differentiation were used (*z*-scored mean TPM). The number of clusters was chosen as the minimal value identifying at least one cluster with maximal expression at each day of differentiation, including day 0. Correlations with developmental gene expression were made by directly plotting the log2FC reported in a previous study using scNMT-seq (single-cell nucleosome, methylation and transcription sequencing) around gastrulation of mouse embryos (Argelaguet et al., 2019). Additional details are available in supplementary Materials and Methods.

### Chromatin analyses

After trypsinisation, ESCs were cross-linked and nuclei isolated and sonicated using a Covaris M220. Fragmented chromatin (typically 200-600 bp) was used for ChIP using the antibodies listed in Table S2. ChIP and the corresponding Input samples were analysed by qPCR using the primers listed in Table S2. Additional information is available in supplementary Materials and Methods.

## Supplementary Material

Supplementary information

Reviewer comments
