# Supplementary material for: H3K9 tri-methylation at Nanog times differentiation commitment and enables the acquisition of primitive endoderm fate
Source: Development. 2022 Sep 9;149(17):dev201074. doi: 10.1242/dev.201074 (PMC9482333; doi:10.1242/dev.201074)
Supplement: Reviewer comments [file develop-149-201074_review_history.pdf]

H3K9 tri-methylation at Nanog times differentiation commitment and enables the acquisition of primitive endoderm fate

Agnès Dubois, Loris Vincenti, Almira Chervova, Maxim V. C. Greenberg, Sandrine Vandormael-Pournin, Déborah Bourc'his, Michel Cohen-Tannoudji and Pablo Navarro
DOI: 10.1242/dev.201074

Editor: James Briscoe

Review timeline

Submission to Review Commons:	7 July 2021
Submission to Development:	28 June 2022
Editorial decision:	25 July 2022
First revision received:	3 August 2022
Accepted:	4 August 2022

Reviewer 1

Evidence, reproducibility and clarity

Summary:

In this manuscript, Dubois and colleagues examine the chromatin modifications of an 8kb region surrounding the Nanog locus. They identify H3K9me3 as enriched in a region between the Nanog enhancer and promoter. H3K9me3 deposition in this region is higher in conditions where Nanog mRNA is lower or heterogenous (FCS + LIF vs 2i + LIF, etc). They proceed to delete 1.8kb within this region (delta-K9 cells) and characterise the effect of the deletion. They show that delta-K9 cells show more homogenous Nanog expression, as well as delayed or impaired differentiation as shown by colony formation assays and gene expression in two different differentiation paradigms.

Major comments:

The authors characterise the chromatin status of the region as well as the effects of the deletion in detail and the data is very convincing. Given the inability of delta-K9 cells to turn off Nanog, the defects in differentiation make sense. However, I am less convinced about the interpretation of the data given the evidence presented, as outlined below:

1. The narrative that the defects observed are a result of loss of H3K9me3 is not fully supported by the data. In the tile and text, the authors imply that the defects in commitment/differentiation associated with the delta-K9 deletion are due to the loss of H3K9me3. However, deletion of 1.8kb will also bring the Nanog enhancer and promoter closer together, potentially leading to increased/more efficient contact and higher expression of Nanog/delayed or impaired Nanog downregulation. Equally possible is that the 1.8kb deletion results in loss of transcription factor binding sites etc. Either of these could explain most of the results observed (retention of Nanog expression, delayed/impaired differentiation). Moreover, the authors also noted that H3K9me3 deposition occurs after Nanog expression is turned off (p4, fig 3C), which suggests the modification is secondary to transcriptional status and not directly/immediately involved in the regulation of Nanog expression. Since a central claim of the paper is that the delta-K9 deletion effects are due to loss of H3K9me3, this should be independently validated. One potential way to exclude that the defects observed are due to the distance or presence of the H3K9me3 domain would be to insert a random 1.8kb fragment in the IR region or to use a KDM4-inactiveCas9 fusion targeted to the IR region to selectively remove H3K9me3 without changing the genomic sequence.

© 2022. Published by The Company of Biologists under the terms of the Creative Commons Attribution License (https://creativecommons.org/licenses/by/4.0/).

2. ERK-dependence. Both in the abstract and text the authors imply that the deposition of H3K9me3 is dependent on ERK ["While in undifferentiated ES cells H3K9me3 at Nanog depends on ERK activity, in somatic cells it becomes ERK independent." (abstract); "Moreover, and in contrast to ES cells, the inhibition of ERK in MEFs did not abolish H3K9me3 at Nanog, which remained robustly enriched (Fig.3A)." and "...H3K9me3 at Nanog is liberated from its strict dependency on ERK." (p3)]. The text comes across as suggesting that ERK regulates H3K9me3, which in turn regulates Nanog heterogeneity. None of the experiments excludes the possibility that ERK controls Nanog expression, and the H3K9me3 deposition a result of Nanog expression status. The experiments in somatic cells further support this: adding PD does not result in changes in Nanog expression and therefore H3K9me3 deposition. Statements such as "While in undifferentiated ES cells H3K9me3 at Nanog depends on ERK activity" appear somewhat misleading. Since FCS+LIF cultures are heterogenous for Nanog expression (and the authors show that only Nanog-negative cells show enrichment in H3K9me3), inhibition of ERK increases (and/or selects for) Nanog expression so that most cells are Nanog positive and therefore H3K9me3-low. I suggest rewriting the statements of causation.

3. Specific defect in primitive endoderm differentiation. I would suggest caution when interpreting the results from the directed differentiation (Figure5 and "Yet, the highest consequences affect genes normally upregulated in the primitive endoderm; remarkably, the lack of H3K9me3 at Nanog is incompatible with differentiation along this lineage." -discussion). The delta-K9 cells completely fail to downregulate Nanog. This is perhaps not surprising for cells that express higher Nanog than normal and are placed in culture conditions that contain both CH and LIF. Therefore, the defect might arise from failure to exit pluripotency/initiate differentiation rather than be specific to primitive endoderm differentiation.

4. "However, while Nanog expression continued to decrease during differentiation of wild-type cells, we observed that K9 clones displayed a stabilisation of low Nanog expression after the sharp decrease occurring during the first 2 days (Fig.3C), despite an efficient differentiation (Fig.S3A)." (p4). This observation is interesting. Does it imply that all cells retain low Nanog expression and upregulate differentiation markers at the same time or that Delta-K9 cells contain a mix of cells? It would be useful to perform immunostainigs to quantify the percentage of Nanog positive cells and determine if differentiation markers (1 or 2 should be enough) are co-expressed with Nanog.

Minor comments:

- qPCR axis. It would be more helpful if the axis clearly indicated what each axis shows instead of "Nanog". It would also be more clear if all results were presented as relative to control gene rather than normalised to 1 to get a better idea of the expression in WT vs delta-K9 cells.
- Figure 4C, last sample label reads PRE E7.5.
- "While naïve pluripotency genes (Esrrb, Klf4, Prdm14, Rex1) showed a less drastic downregulation, mimicking Nanog expression, differentiation markers
(Fgf5, Dnmt3b, Otx2, Wnt3) showed delayed dynamics." There is a lot of overlap between the points. With the exception of Dnmt3b it is unclear that any of the other facts are significantly delayed.
- Primitive endoderm vs definitive endoderm. All of the genes selected to identify primitive endoderm are also expressed in definitive endoderm. It would strengthen the point to show markers exclusively expressed in a lineage and not the other (e.g. Sox7, Pdgfra) or picking examples from the clusters used in Figure 4. Could the authors also clarify whether there is overlap in the genes that belong to the 4 embryo gene clusters of if they are unique?
- "In agreement with the low upregulation of Nanog in _ K9 cells, we observed a nearly insignificant increase in self renewal efficiency, as determined by clonal assays." (p2). The meaning of this sentence is unclear.
- Figure 2C, please include x axis label.
- "Moreover, cellular outgrowths derived from _ K9 EBs also exhibited obvious differences compared to those derived from wild-type EBs, with less apparent multi-lineage differentiation (Fig.S4A)." (p4). Figure S4A shows brightfield images of EBs and EB outgrowths. It is difficult to determine from these how the authors conclude about multi-lineage differentiation.
- "the regulation of Nanog appears to involve an intermediary state where
H3K9me3 is already established but not yet fixed." (discussion) and "This mitotically-stable and ERK-dependent state of H3K9me3 confers to Nanog silencing the required stability to be inherited and,

© 2022. Published by The Company of Biologists under the terms of the Creative Commons Attribution License (https://creativecommons.org/licenses/by/4.0/).

at the same time, sufficient flexibility to revert back to transcriptional activity." (discussion). For this to be true, Nanog-negative H3K9me3-marked cells would need to re-activate Nanog and lose H3K9me3. However, the authors present no evidence of this "flexibility". Since H3K9me3 deposition lags behind Nanog downregulation, it is possible that Nanog-low/H3K9me3-low cells to are able to reactivate Nanog and expand, while Nanog-low/H3K9m3-high results in stable repression.
-"Since the deletion of the region harbouring H3K9me3 leads to a minor increase of NANOG expression, it was not expected to block differentiation. After all, upon the collapse of the pluripotency network triggered by differentiation signals, Nanog would lose most of its activators and be downregulated, as we observed." (discussion). This statement is not really consistent with the results section where the authors noted that "naïve pluripotency genes (Esrrb, Klf4, Prdm14, Rex1) showed a less drastic downregulation, mimicking Nanog expression...".

Significance

The study is timely and interesting as it touches a number of topics: stable inheritance of chromatin modifications, link between signalling, chromatin status and transcription, how these influence differentiation, and heterogeneity. Therefore, this report would be of interest to a wide community.
Expertise: ES cell biology, differentiation, early embryology, control of gene expression, signalling.

Reviewer 2

Evidence, reproducibility and clarity

In this manuscript, Dubois et al. interrogate the mechanisms underlying the epigenetic regulation of Nanog in mouse ES cells. One of the key questions in stem cell biology is how cell fate decisions are controlled. Perhaps the most important of these decisions for a stem cell is the choice between maintaining the stem cell state or initiating differentiation. It is well known that the transcription factor Nanog is one of the key components of the gene network that maintains the ES cell state, and its loss marks cells that are prone to differentiate into the Primitive Endoderm lineage (Gata6+). How some cells within the early embryo start becoming Nanog-low and turn up the Primitive Endoderm program still remains unknown. Interestingly, the Nanog expression state displays this heterogeneity even among cultured ES cells (in LIF). Intringuingly, this expression state can be inherited from parent to daughter cells with relative precision for a number of cell divisions before some cells can start turning it off. How this switch-like behavior ("memory") is orchestrated remains a mystery and this is what Dubois et al. have began to unveil in this manuscript. Since nucleosomal H3 subunits are known to be heritable among daughter cells during mitosis, Dubois et al. focused on H3 modifications which are known to mark gene transcriptional activity. Looking at a panel of these modifications, they found H3K9me to be significantly enriched in a specific region upstream of the Nanog promoter. By removing this region, they proved its role in regulating heterogeneity Nanog, and although not directly shown by the authors, perhaps also related to the Nanog expression memory. Since H3K9me was the only chromatin silencing modification found within this region, the authors conclude that H3K9me acquisition within this element might be one of the earliest regulatory hallmarks responsible for generating the memorizable Nanog-low cell state that is prone to differentiate into PrE.

Even though I am not an expert in Nanog regulation or ES cell biology, it was easy for me to appreciate that this is a very thorough study attempting to obtain some important insights on the mechanisms of stem cell fate decisions. Perhaps due to my distance to the field, I could verify that readers not familiar with the tools and techniques should be able to read this manuscript and distill the key insights with little background other than what is presented in the introduction.

The figures and supplementary data are well-presented and concise enough to understand the key experimental evidence that supports the conclusions. The methods are also described in detail, enough to allow anyone with minimal ES cell culture knowledge and basic molecular biology training to reproduce the large majority of results. Perhaps the statistical analyses are a bit more obscure and hard to interpret from looking only at the figures or figure legends. Only a few of these data tables are present and in some cases, the number of experiments performed is not easy to

© 2022. Published by The Company of Biologists under the terms of the Creative Commons Attribution License (https://creativecommons.org/licenses/by/4.0/).

obtain. In addition, it is unclear if any statistical approach was followed to formally challenge some of the most important hypotheses. As an example, in Fig. 2C, the Nanog levels of WT and K9-KO cells are compared using a histogram plot, and it is claimed that Nanog heterogeneity is lost in K9-KO cells compared to WT. At least a simple statistical comparison of variation measures (i.e. compare the variance across n=5 independent culture+staining experiments) should be included. Although I cannot be thorough here, I have noted that this type of issue showed up on more than one occasion: i.e., authors stating a conclusion but with statistical analysis unclear or not shown (I also couldn't find any details on statistical methods in the Supplementary Methods). While this is the most important major concern for me, I do think that it is easy to fix, and should take authors a reasonable amount of time to take care of it. As a more challenging aspect that would improve the manuscript, I believe that deleting the K9me region in Nanog-GFP cell lines would help validate the heterogeneity conclusions drawn just from IF studies.

My other, more "minor" concerns are presented in bullet-point style:

1) Without 2i, Nanog+ cells seem to have almost normal levels of H3K9me in the intermediate region element that is identified in this study (about 2% of IP, based on the y-axis units). Sure, in comparison, Nanog- cells have much higher levels of H3K9me, but Nanog+ cells seem to have quite a bit of K9me still... I'm not sure whether something so arbitrary as "% of IP material" can be assessed in such absolute terms as I'm doing here. But, what do authors make of this result, regarding their conclusions about Nanog epigenetic memory and timing of differentiation... Is it possible that K9me begins to be deposited in Nanog+ cells, but then the timing involves further deposition of K27me3?

2) Deletion of the IR element (ΔK9) leads to higher steady-state Nanog expression. Is the difference between WT and ΔK9 cells lost in the presence of the ERK inhibitor?

3) How necessary is this element for ERK-related effects on ES cells? For example, do ΔK9 cells with Chir+LIF resemble 2i+LIF WT cells?

4) A major conclusion seems to be the loss of Nanog heterogeneity upon K9me element deletion. Do any other experiments support this "loss of heterogeneity"? Perhaps performing colony (or single-cell culture) assays and staining with Nanog IF would be helpful?

5) In fig 2A, there seem to be much lower H3K9me levels across the entire Nanog gene body, and not just in the deleted región suggesting a deterministic role for this region (perhaps as an initiator of gene silencing)... What happens with LIF withdrawal? Does K9me appear gradually, even in spite of the deleted element? Is the silencing just based on K27me3 accumulation (independent of K9me)? -

6) A big assumption of the conclusions is that K9me is the most important thing that happens to that region, but this is based only on the analysis of a panel of ChIP with anti-modified-histone antibodies... Does any transcription factor bind there?

7) How do the authors propose that ERK actually regulates this epigenetic mechanism? Additional evidence would be helpful here. For instance, how fast can ERK inhibition cause loss of H3K9me?

8) An aspect of Nanog epigenetic regulation by ERK still eludes my conscience: Say you take Nanog negative cells and positive cells and separate them by FACS. After separating, both populations are treated with the PD inhibitor. Then PD is washed out. Do previously Nanog-negative cells turn Nanog down faster than the positive ones? This might help clarify whether just ERK or other additional pathways might be contributing to the Nanog memory function.

9) Plasticity of Nanog state: Authors state that Nanog cannot be turned on by PD after a certain differentiation switch has occurred, and they show that ERK inhibition does not lead to K9me changes in the Nanog intermediate regions within MEFs. But what about differentiating ES cells? Authors could try PD treatments 1-3 days after LIF removal (instead of putting it on MEFs) - At which point during differentiation is the decision to turn into XEN reversible?

© 2022. Published by The Company of Biologists under the terms of the Creative Commons Attribution License (https://creativecommons.org/licenses/by/4.0/).

Significance

The advance presented is mostly incremental instead of paradigm-shifting.
However, due to the importance of understanding Nanog regulation in pluripotency and the lack of mechanistic insights about its epigenetic regulation, this is a highly impactful study.
Previously published knowledge has focused more on gene regulatory networks, other chromatin modifications, or broad unbiased whole-transcriptome analyses. Instead, this study presents a more nuanced, focused, and precise dissection of the Nanog epigenetic regulatory elements. As such, from a biologist's perspective, it is interesting as it shows a very interesting model for how stem cell epigenetic memory might actually be regulated. Yet, from a medical perspective, it should still be an interesting read for those looking for ways to manipulate Nanog expression or other genes in ES cells exiting quiescence (either for therapy or engineering).

Referee Cross-commenting

I generally agree with the other 2 reviewers. I think that the experiment with dCas9 fused to epigenetic regulators that both reviewers seem to suggest might be very interesting to perform, although I believe that it could be very challenging (and still imperfect in many other ways). A bit more bioinformatic analysis of the K9me region and additional probing of its dynamics, plus some additional angles of evidence of heterogeneity, could be enough proof. Granted, if important regulatory TF motifs are in the deleted region, or, if there are other lines of evidence that the region might be involved in Nanog activation by other means, then this will require either toning down some important conclusions or performing more specific experiments (either performing smaller deletions or using dCas9-fused histone modifiers, indeed).

Reviewer 3

Evidence, reproducibility and clarity

In this study, Dubois et al investigate the underlying mechanisms that enable (naïve pluripotent) cells to dynamically transition between distinct gene expression 'states'. Further they ask how such regulated heterogeneity is linked with lineage diversification and fate-decisions during cellular commitment phases. Using dynamic Nanog regulation as a paradigm, the authors initially map chromatin changes associated with expression states, and subsequently focus on a genomic region that acquires H3K9me3 coincident with expression status and/or cell type, and which shows a degree of heritability. They go onto to genetically delete this putative 'epigenetic' control region and elegantly show through a sequential molecular and cellular assay that it is linked with Nanog dynamics/stable silencing and ultimately cell fate. This implicates H3K9me3 as an epigenetic mechanism underlying cellular heterogeneity, which in turn feeds into acquisition of lineage identity during differentiation, particularly primitive endoderm in this case.

This is an elegantly -designed and -performed study that carefully maps molecular events and mechanisms that have broad relevance for understanding development. Moreover, the study emphasises how multiple mechanisms integrate differentially to regulate gene expression programmes in distinct contexts (e.g. the switchable influence of ERK signaling on H3K9me3 in pluripotent vs differentiated cells). This feeds into general concepts of what constitutes 'epigenetic' control. The study is well-written and cohesive. Nevertheless, I have some comments on whether the clear readouts the authors observe can be attributed specifically to H3K9me3 based on the perturbations performed. Given this is central to their conclusions, further exploration of the direct functional importance of chromatin states per se (H3K9me3 or other) for Nanog heterogeneity/dynamics could be useful to strengthen conclusions. Some comments and suggestions along these lines are made below. With modifications and clarifications, I support this manuscript for publication.

Major comments.

1.) Distinguishing between genetic and epigenetic effects.
The authors identify a region of the upstream Nanog promoter based on its strong enrichment of

© 2022. Published by The Company of Biologists under the terms of the Creative Commons Attribution License (https://creativecommons.org/licenses/by/4.0/).

H3K9me3 specifically when Nanog is heterogenous and/or silenced. They hypothesized this acquired H3K9me3 could be important for expression dynamics and indeed it correlates well. To functionally test this the specific region is deleted with CRISPR and these cells are referred to as Delta-"K9". A concern here is that whilst indeed the region of H3K9me3 has been removed, so has the underlying DNA sequence. In principle, the effects observed downstream (Nanog expression, cell fate skewing etc) could reflect the absence of key TF, repressor, or insulator motifs within this region, or perturbation to genomic contact sites, rather than absence of H3K9me3 per se.

I think this needs at least further clarification/discussion in the text since the manuscript refers solely to these cells as lacking H3K9me3 at the assayed region. The discrepancy should ideally be tested experimentally to draw sturdy conclusions. For example, by using existing dCas9-based epigenetic editing tools to perturb chromatin states without genetic manipulation. Here, use of a KRAB (in Nanog positive) or VPR (in Nanog negative cells) could provide some information on a causal effect of depositing/perturbing H3K9me3 states directly (well upstream of Nanog TSS). Even better would be to specifically deposit of remove H3K9me3 using a programmable (de)methylase. Taking a less precise but more tractable approach could involve siRNA of the relevant H3K9me3 methylase and/or inhibitors to link Nanog activity with global H3K9me perturbation.

More generally, more information on the function/attributes of the deleted segment would be valuable. What TF motifs are there? What loci does it loop to in contact maps? Is it CpG-poor/rich? What features seem to underlie its H3K9me3 targeting? etc. Indeed, it could be interesting in future to make more precise genetic perturbation of the locus (inversion, specific motif deletion etc) to dissect its key control function.

2.) Function of other chromatin marks at the regulatory region.
The authors focus on H3K9me3 since it shows the greatest discrepancy between 2i (active) and Serum/Lif (heterogenous) ESC, and correlates well with read-outs. Nevertheless, H3K4me2 and histone acetylation at the same genomic locus also shows this correlation (inversely) (Fig 1B). It could be that changes in these chromatin states are upstream and/or causal of the observed changes in H3K9me3. In other words can the authors comment on the possibility that Nanog activity is linked with dynamic deposition of activating marks such as acetylation, which may or may not in turn be linked with the observed H3K9me3 dynamics.

3.) Function of H3K9me3.
Whilst the authors convincingly demonstrate that when the H3K9me3 harboring region is deleted it leads to a number of important molecular and cellular consequences (Nanog expression dynamics, and cell identity/fate), it is unclear how H3K9me3 deposition >1kb from the TSS might exert such function. Indeed, in FCS+LIF ESC or during differentiation H3K9me3 still does not encroach near the TSS. It would be interesting to discuss or test the potential molecular mechanisms by which distal H3K9me3 functions, given the important influence it seems to have on locking down Nanog silencing. Does it affect chromatin compaction, contacts, TF binding?

4.) Statistics. There is a general absence of statistics applied. For clarity, it would be useful to add appropriate statistical tests to quantitative measurements throughout (e.g. Fig 1A, 2B, 2D, 3C, 3D, 4A, 4D).

Jamie Hackett; assignment accepted: 26 July 2021; completed: 05 Aug 2020.
My standard policy is to sign and date ALL peer review reports, irrespective of my comments or recommendations. Further communication related to this should be via the editorial office. Please do not remove this note.

Significance

The study will provide a valuable insight to the community and provide an intriguing cellular example of how and when gene regulation becomes 'epigenetic' - independent of inducing signals and reliant on pre-existing state. This feeds into understanding how cell fates become allocated and how cells acquire competence to respond to different cues during development.

© 2022. Published by The Company of Biologists under the terms of the Creative Commons Attribution License (https://creativecommons.org/licenses/by/4.0/).

Author response to reviewers' comments

Point by point response to the reviewers' comments.

General observations and information to all referees.

We would like to acknowledge our three referees for what we think is a very fair evaluation of our work. Most, if not all comments and criticisms were justified. We have therefore made considerable efforts to address them. However, we would like to clarify that the approach we followed was somewhat orthogonal to the specific requests made by the referees. Indeed, prompted by some comments (as described below), we identified the underlying molecular mechanism explaining our observations. Briefly, we now show that DNA methylation, known to be dependent on ERK activity in ES cells, and ZFP57 binding, known to trigger H3K9me3, are at the bottom of the mechanisms leading to our observations. Through the examination of appropriate knock-outs, we believe that the most important criticisms have been fully addressed. As this data will be used to reply to different comments, we describe it here (Fig.R1) and have fully included it in the manuscript as Fig.6. In the revised manuscript, all text changes are shown in red.

Briefly, we now show that the *Nanog* region enriched for H3K9me3 encompasses two motifs for ZFP57, a TF that has been already shown to recruit H3K9 methylases in ES cells. To bind, ZFP57 requires a methylated CpG: satisfactorily, both are methylated in cells cultured in FCS+LIF but not in 2i+LIF (Fig.R1A,B). Based on these analyses, we explored ZFP57 binding in FCS+LIF, 2i+LIF and DNMT1/3a/3b TKO cells (using a cell line generated by D. Bourc'his and M. Greenberg, now authors of our manuscript – the characterisation of the line is described in Fig.S7). Our ChIP-qPCR analysis confirms binding of ZFP57 at the expected region in wild-type cells cultured in FCS+LIF exclusively (Fig.R1C). In line with this, we further show that H3K9me3 enrichment is highly correlated to ZFP57 binding as it is lost in DNMT1/3a/3b TKO cells as well as in previously described ZFP57 KO (ZKO) ES cells (PMID:27257070; Fig.R1D). These results do not only provide a clear molecular scenario where the ERK-CpG.CH3-ZFP57-H3K9me3 axis mechanistically substantiates our work, they also address several major concerns of our referees, especially but not exclusively whether the deleted DNA sequence has additional roles beyond H3K9me3. Indeed, these two mutants have a wild-type *Nanog* locus but they display reduced NANOG heterogeneity (Fig.R1E) and altered primitive endoderm differentiation (Fig.R1F,G).

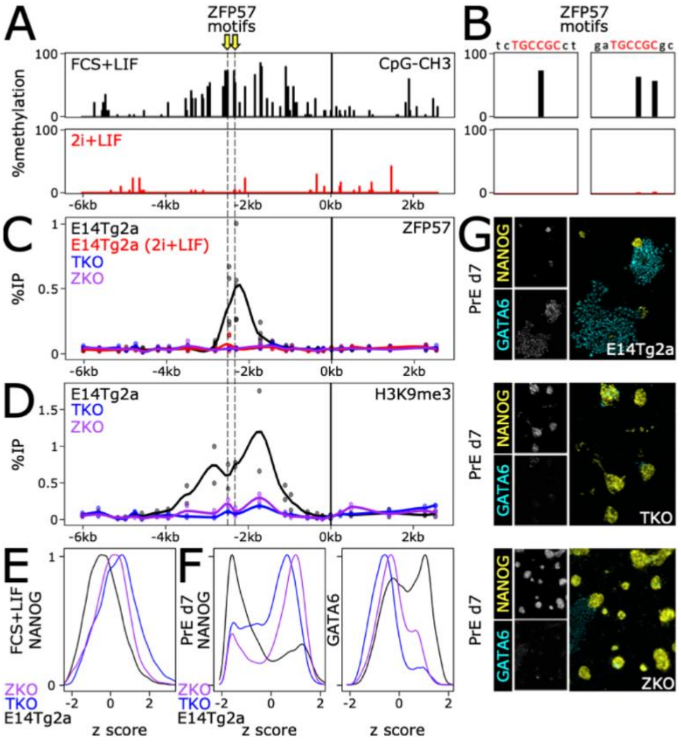

© 2022. Published by The Company of Biologists under the terms of the Creative Commons Attribution License (https://creativecommons.org/licenses/by/4.0/).

Fig.R1: DNA methylation and ZFP57 trigger H3K9me3 at *Nanog*. **(A)** DNA methylation at *Nanog*. The 2 ZFP57 motifs identified over the H3K9me3-enriched region are shows. **(B)** DNA methylation at the two ZFP57 motifs. **(C)** Analysis of ZFP57 binding by ChIP in wt (E14Tg2a), triple DNMT1/3a/3b knock-out (TKO) and ZFP57 knock-out (TKO) undifferentiated ES cells. **(D)** Analysis of H3K9me3 in wt, TKO and ZKO undifferentiated ES cells. **(E)** Analysis of NANOG expression in wt, TKO and ZKO undifferentiated ES cells. **(F)** Analysis of NANOG and GATA6 expression in wt, TKO and ZKO cells subject to a direct PrE differentiation protocol. **(G)** Representative images of PrE-differentiated cells stained for NANOG and GATA6.

Hence, we would like to thank all three referees for triggering a whole new line of investigation that has helped us make what we believe is a much stronger and relevant contribution to the field. Below, a detailed response is given to every comment raised. All data supporting our new claims is presented in this document and a substantial part has been included in our new version of the manuscript, as indicated where and when appropriate.

Reviewer #1 (Evidence, reproducibility and clarity (Required)):

Summary:
In this manuscript, Dubois and colleagues examine the chromatin modifications of an 8kb region surrounding the Nanog locus. They identify H3K9me3 as enriched in a region between the Nanog enhancer and promoter. H3K9me3 deposition in this region is higher in conditions where Nanog mRNA is lower or heterogenous (FCS + LIF vs 2i + LIF, etc). They proceed to delete 1.8kb within this region (delta-K9 cells) and characterise the effect of the deletion. They show that delta-K9 cells show more homogenous Nanog expression, as well as delayed or impaired differentiation as shown by colony formation assays and gene expression in two different differentiation paradigms.

We acknowledge that the referee has made a precise and concise summary of our work. We would like to clarify, however, that three different differentiation paradigms were used: N2B27 differentiation, Embryoid Bodies and Primitive Endoderm directed differentiation.

Major comments:

The authors characterise the chromatin status of the region as well as the effects of the deletion in detail and the data is very convincing. Given the inability of delta-K9 cells to turn off Nanog, the defects in differentiation make sense. However, I am less convinced about the interpretation of the data given the evidence presented, as outlined below:

We thank the referee for the positive comment made about our data, even if we understand they disagree with some of our interpretations. As explained below, we believe this is largely based on specific misunderstandings that call for some rewording of key parts of the main text.

1. The narrative that the defects observed are a result of loss of H3K9me3 is not fully supported by the data. In the tile and text, the authors imply that the defects in commitment/differentiation associated with the delta-K9 deletion are due to the loss of H3K9me3. However, deletion of 1.8kb will also bring **the Nanog enhancer and promoter closer together, potentially leading to increased/more efficient contact and higher expression of Nanog/delayed or impaired Nanog downregulation. Equally possible is that the 1.8kb deletion results in loss of transcription factor binding sites etc**. Either of these could explain most of the results observed (retention of Nanog expression, delayed/impaired differentiation). Moreover, the authors also noted that H3K9me3 deposition occurs after Nanog expression is turned off (p4, fig 3C), which suggests the **modification is secondary to transcriptional status and not directly/immediately involved in the regulation of Nanog expression.** Since a central claim of the paper is that the delta-K9 deletion effects are due to loss of H3K9me3, this should be independently validated. One potential way to exclude that the defects observed are due to the distance or presence of the H3K9me3 domain would be to insert a random 1.8kb fragment in the IR region or to use a KDM4-inactiveCas9 fusion targeted to the IR region to selectively remove H3K9me3 without changing the genomic sequence.

© 2022. Published by The Company of Biologists under the terms of the Creative Commons Attribution License (https://creativecommons.org/licenses/by/4.0/).

We agree with the referee that the nature of our deletion opens up the possibility of additional confounding factors influencing *Nanog* behaviour. We have now added new data analysing cells that lack H3K9me3 at *Nanog* but that remain WT at the *Nanog* locus. These cell lines are Triple Negative KO *Dnmt1/3a/3b* (TKO) and *Zfp57* KO (ZKO) cells (Fig.R1). Both cells phenocopy key events described in our mutants, such as reduced heterogeneity and impaired PrE differentiation. We hope this data will mitigate the point made by this referee even if we followed a different strategy than the two that were proposed. Nevertheless, as we agree that it is impossible to fully rule out additional effects mediated by the region independently of H3K9me3, we have now more carefuly stated on the main text such limitations: "***While the existence of other regulations mediated by the deleted region*** ***cannot be formally excluded****, the fact that DNMTs and ZFP57 knock-outs (which have a wild-type Nanog locus) phenocopy the loss of NANOG heterogeneity and the alteration of primitive endoderm differentiation, suggests that H3K9me3 plays a major role."* on page 13 of the new version.

We have also clarified that H3K9me3 deposition does not always "*occur after Nanog expression is turned off*", as this referee understood. For instance, in the Nanog-GFP positive cells (TNG) already shown in the original manuscript (Fig.1D of the new version), we still detect H3K9me3 albeit at lower levels than in negative cells. To further reinforce this important point, we have added H3K9me3 ChIP-qPCR data in *Nanog*-GFP reporter cells cultured in the presence of Puromycin. In these cells, the selection cassette is linked to the *Nanog*-GFP allele and Puromycin (Fig.R2A) selects for virtually 100% of cells expressing *Nanog* (PMID:18097409 & 23178592). As shown in Fig.R2B, these cells still display H3K9me3 at *Nanog*. This notion was correctly interpreted by Reviewer 2 (minor point 1), who asks why then H3K9me3 is not always associated with *Nanog* downregulation – we direct this referee to our response to Reviewer 2 should they consider this matter is of interest. This data is now added as Fig.S1A,B and described on page 5: "*H3K9me3 was also found present, albeit at low levels, in Nanog-positive cells, obtained either by FACS (Fig.1D) or by taking advantage of a puromycin selection cassette linked to the Nanog-GFP allele (Fig.S1A,B)*".

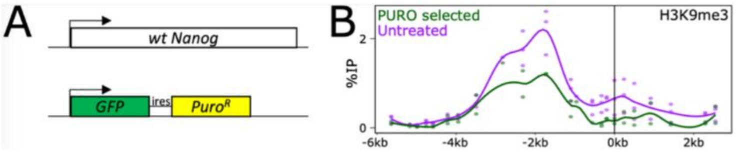

Fig.R2: Presence of H3K9me3 in actively expressing ES cell populations.
(A) Schematic of Nanog alleles in TNG cells. **(B)** Analysis of H3K9me3 in PURO selected or in untreated populations.

2. ERK-dependence. Both in the abstract and text the authors imply that the deposition of H3K9me3 is dependent on ERK ["While in undifferentiated ES cells H3K9me3 at Nanog depends on ERK activity, in somatic cells it becomes ERK independent." (abstract); "Moreover, and in contrast to ES cells, the inhibition of ERK in MEFs did not abolish H3K9me3 at Nanog, which remained robustly enriched (Fig.3A)." and "...H3K9me3 at Nanog is liberated from its strict dependency on ERK." (p3)]. The text comes across as suggesting that ERK regulates H3K9me3, which in turn regulates Nanog heterogeneity. **None of the experiments excludes the possibility that ERK controls Nanog expression, and the H3K9me3 deposition a result of Nanog expression status**. The experiments in somatic cells further support this: adding PD does not result in changes in Nanog expression and therefore H3K9me3 deposition. Statements such as "While in undifferentiated ES cells H3K9me3 at Nanog depends on ERK activity" appear somewhat misleading. Since FCS+LIF cultures are heterogenous for Nanog expression (**and the authors show that only Nanog-negative cells show enrichment in H3K9me3**), inhibition of ERK increases (and/or selects for) Nanog expression so that most cells are Nanog positive and therefore H3K9me3-low. I suggest rewriting the statements of causation.

We apologise if the main text led this referee to some misunderstandings, which we have already clarified in the previous point: we now provide more direct evidence that H3K9me3 deposition is not "*a result of Nanog expression status*" and that not "*only Nanog-negative cells show enrichment in H3K9me3*". However, the essence of this point, namely that the causative effects were unclear in our previous manuscript, remains valid. We hope that the link now established with DNA methylation and ZFP57 binding will contribute to clarify this issue: ERK inhibition is known to abolish DNA methylation in ES cells

© 2022. Published by The Company of Biologists under the terms of the Creative Commons Attribution License (https://creativecommons.org/licenses/by/4.0/).

(https://doi.org/10.1101/2021.11.18.469000), leading to a lack of ZFP57 binding and, therefore, a loss of H3K9me3 enrichment (Fig.R1A-D). Accordingly, in both DNMT TKO and ZFP57 KO cells, NANOG expression is more homogeneously expressed (Fig. R1E). This data is now shown as Fig.6 and fully described on pages 10-11 of the current manuscript.

3. Specific defect in primitive endoderm differentiation. I would suggest caution when interpreting the results from the directed differentiation (Figure5 and "Yet, the highest consequences affect genes normally upregulated in the primitive endoderm; remarkably, the lack of H3K9me3 at Nanog is incompatible with differentiation along this lineage." -discussion). **The delta-K9 cells completely fail to downregulate Nanog. This is perhaps not surprising for cells that express higher Nanog than normal and are placed in culture conditions that contain both CH and LIF. Therefore, the defect might arise from failure to exit pluripotency/initiate differentiation rather than be specific to primitive endoderm differentiation**.

In contrast to the claim made by this referee, the defects we observe cannot arise "*from failure to exit pluripotency/initiate differentiation*". We showed indeed in the original manuscript that the ΔK9 cells differentiate efficiently in different paradigms we explored but not in directed differentiation towards PrE. In fact, the referee quotes one of our sentence ("*the highest consequences affect genes normally upregulated in the primitive endoderm*" on page 13 of the current ms) in the context of a PrE differentiation when in reality it describes the effects observed in standard EB differentiation assays. To avoid any misunderstanding by other readers we have now added text to our discussion more directly stating our observations and conclusions, on page 13: "*using multilineage protocols we observed delayed commitment and altered differentiation into all germ layers of cells lacking the H3K9me3-enriched region. Yet, the highest consequences affect genes normally upregulated in the primitive endoderm, an observation that was fully confirmed by their incapacity to efficiently differentiate into primitive endoderm using a directed differentiation protocol.*"

We would also like to clarify that the lack of induction of PrE markers is not specific to directed differentiation, where we acknowledge the presence of CH and LIF may add complexity to the regulation of *Nanog*. We also observe this in EB formation assays, where both CH and LIF are absent.

4. "However, while Nanog expression continued to decrease during differentiation of wild-type cells, we observed that K9 clones displayed a stabilisation of low Nanog expression after the sharp decrease occurring during the first 2 days (Fig.3C), despite an efficient differentiation (Fig.S3A)." (p4). This observation is interesting. Does it imply that all cells retain low Nanog expression and upregulate differentiation markers at the same time or that Delta-K9 cells contain a mix of cells? It would be useful to perform immunostainigs to quantify the percentage of Nanog positive cells and determine if differentiation markers (1 or 2 should be enough) are co-expressed with Nanog.

This is an important point that we have experimentally addressed by immunostaining of differentiating cells (Fig.R3). By d7 of 2i+LIF withdrawal, we observe a rather homogeneous reduction of NANOG expression in our mutant cells, which is less drastic than in wild-type cells. We conclude from this that most cells "*retain low Nanog expression*" rather than the other possibility of a small subset of cells displaying high levels of NANOG. This has been added as Fig.S4 and commented on page 7: "*Immuno-fluorescence analyses further indicated that the retention of low but measurable NANOG expression affected the vast majority of ΔK9 cells (Fig.S4).*".

© 2022. Published by The Company of Biologists under the terms of the Creative Commons Attribution License (https://creativecommons.org/licenses/by/4.0/).

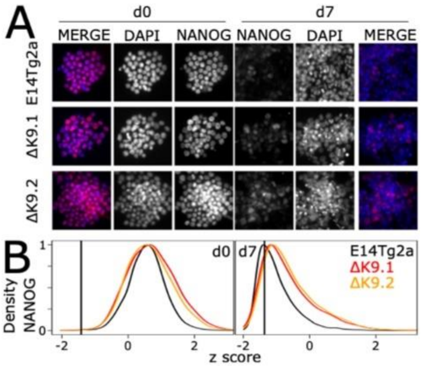

Fig.R3: Global retention of low NANOG levels in differentiating ΔK9 ES cells. **(A)** Illustrative staining of NANOG. **(B)** Quantitative analysis of NANOG expression.

Minor comments:

- qPCR axis. It would be more helpful if the axis clearly indicated what each axis shows instead of "Nanog". It would also be more clear if all results were presented as relative to control gene rather than normalised to 1 to get a better idea of the expression in WT vs delta-K9 cells.

We have now amended the Y axis labels to state the specific metric used. When we normalise to 1 it is always relative to control, this has been clarified across legends.

- Figure 4C, last sample label reads PRE E7.5.:

This has been corrected to PRE E4.5.

- "While naïve pluripotency genes (Esrrb, Klf4, Prdm14, Rex1) showed a less drastic downregulation, mimicking Nanog expression, differentiation markers (Fgf5, Dnmt3b, Otx2, Wnt3) showed delayed dynamics." There is a lot of overlap between the points. With the exception of Dnmt3b it is unclear that any of the other facts are significantly delayed.

We agree the differences are subtle for *Fgf4, Dnmt3b* and *Otx2*. This has been acknowledged in the text, page 8: "*differentiation markers (Fgf5, Dnmt3b, Otx2, Wnt3) showed* ***slightly*** *delayed dynamics*".

- **Primitive endoderm vs definitive endoderm**. All of the genes selected to identify primitive endoderm are also expressed in definitive endoderm. It would strengthen the point to show markers exclusively expressed in a lineage and not the other **(e.g. Sox7, Pdgfra)** or picking examples from the clusters used in Figure 4. Could the authors also clarify whether there is overlap in the genes that belong to the 4 embryo gene clusters of if they are unique?

There is no gene overlap between the clusters: those were made on our data and their expression interrogated in published embryo datasets. We have also added as Fig.S5B RNA-seq examples of other primitive endoderm markers, including *Sox7* and *Pdgfra* as requested by the referee, shown here as Fig.R4.

© 2022. Published by The Company of Biologists under the terms of the Creative Commons Attribution License (https://creativecommons.org/licenses/by/4.0/).

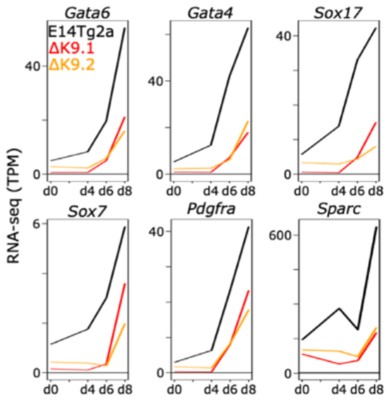

Fig.R4: Expression of (primitive) endoderm markers during EB differentiation.

- "In agreement with the low upregulation of Nanog in _ K9 cells, we observed a nearly insignificant increase in self renewal efficiency, as determined by clonal assays." (p2). The meaning of this sentence is unclear.

NANOG is known to increase self-renewal efficiency when strongly upregulated from ectopic transgenes that are not subject to the endogenous regulations of *Nanog*. We were therefore not expecting that our mutant cells would promote self-renewal given the moderate increase of NANOG we observed and to its effective downregulation upon differentiation. The text has now been clarified on page 6: "*whereas strong ectopic induction of NANOG leads to improved self-renewal (Chambers et al., 2007), the small upregulation of Nanog in ΔK9 cells was associated with a marginal increase in self- renewal efficiency, as determined by clonal assays (Fig.2D).*".

- Figure 2C, please include x axis label.:

This has been implemented.

- "Moreover, cellular outgrowths derived from _ K9 EBs also exhibited obvious differences compared to those derived from wild-type EBs, with less apparent multi-lineage differentiation (Fig.S4A)." (p4). Figure S4A shows brightfield images of EBs and EB outgrowths. **It is difficult to determine from these how the authors conclude about multi-lineage differentiation**.

We understand the comment and have therefore reworded this sentence on page 8: "*cellular outgrowths derived from ΔK9 EBs also exhibited **less morphological typologies** compared to those derived from wild-type EBs, **suggesting** altered multi-lineage differentiation*".

- "the regulation of Nanog appears to involve an intermediary state where H3K9me3 is already established but not yet fixed." (discussion) and "This mitotically-stable and ERK-dependent state of H3K9me3 confers to Nanog silencing the required stability to be inherited and, at the same time, sufficient flexibility to revert back to transcriptional activity." (discussion**). For this to be true, Nanog-negative H3K9me3-marked cells would need to re-activate Nanog and lose H3K9me3**. However, the authors present no evidence of this "flexibility". Since H3K9me3 deposition lags behind Nanog downregulation, it is possible that Nanog-low/H3K9me3-low cells to are able to reactivate Nanog and expand, while Nanog-low/H3K9m3-high results in stable repression.

Since we now more convincingly show that *Nanog*-GFP high/low display quantitative differences in H3K9me3 at *Nanog* (Fig.R2), and we had already shown that ERK inhibition leads to a depletion of H3K9me3, we believe that the statements remain correct. Moreover, the new data added on the functional link with DNA methylation (Fig.R1), which is known to be highly dynamic genome-wide when cells transit from heterogeneous (FCS+LIF) to homogeneous (2i+LIF) NANOG expression states (PMID: 30031774), further shows that the "flexibility" we report is an already established fact in the field, which was however not described for H3K9me3 at *Nanog*.

© 2022. Published by The Company of Biologists under the terms of the Creative Commons Attribution License (https://creativecommons.org/licenses/by/4.0/).

-"Since the deletion of the region harbouring H3K9me3 leads to a minor increase of NANOG expression, it was not expected to block differentiation. After all, upon the collapse of the pluripotency network triggered by differentiation signals, Nanog would lose most of its activators and be downregulated, as we observed." (discussion). This statement is not really consistent with the results section where the authors noted that "naïve pluripotency genes (Esrrb, Klf4, Prdm14, Rex1) showed a less drastic downregulation, mimicking Nanog expression...".

We disagree with the interpretation made by the referee because the deficient silencing of other pluripotency TFs operates only late during differentiation (like for *Nanog*), as shown in the original manuscript. At the beginning of differentiation they are as efficiently downregulated as in wt cells.

Reviewer #1 (Significance (Required)):

The study is timely and interesting as it touches a number of topics: stable inheritance of chromatin modifications, link between signalling, chromatin status and transcription, how these influence differentiation, and heterogeneity. Therefore, this report would be of interest to a wide community. Expertise: ES cell biology, differentiation, early embryology, control of gene expression, signalling.

We thank the referee for the positive evaluation of the significance of our work. We hope that with the clarifications made above and the data added with DNMTs TKO and ZFP57 KO cells the issues that were raised have been largely mitigated.

Reviewer #2 (Evidence, reproducibility and clarity (Required)):

In this manuscript, Dubois et al. interrogate the mechanisms underlying the epigenetic regulation of Nanog in mouse ES cells. One of the key questions in stem cell biology is how cell fate decisions are controlled. Perhaps the most important of these decisions for a stem cell is the choice between maintaining the stem cell state or initiating differentiation. It is well known that the transcription factor Nanog is one of the key components of the gene network that maintains the ES cell state, and its loss marks cells that are prone to differentiate into the Primitive Endoderm lineage (Gata6+). How some cells within the early embryo start becoming Nanog-low and turn up the Primitive Endoderm program still remains unknown. Interestingly, the Nanog expression state displays this heterogeneity even among cultured ES cells (in LIF). Intringuingly, this expression state can be inherited from parent to daughter cells with relative precision for a number of cell divisions before some cells can start turning it off. How this switch-like behavior ("memory") is orchestrated remains a mystery and this is what Dubois et al. have began to unveil in this manuscript. Since nucleosomal H3 subunits are known to be heritable among daughter cells during mitosis, Dubois et al. focused on H3 modifications which are known to mark gene transcriptional activity. Looking at a panel of these modifications, they found H3K9me to be significantly enriched in a specific region upstream of the Nanog promoter. By removing this region, they proved its role in regulating heterogeneity Nanog, **and although not directly shown by the authors, perhaps also related to the Nanog expression memory**. Since H3K9me was the only chromatin silencing modification found within this region, the authors conclude that H3K9me acquisition within this element might be one of the earliest regulatory hallmarks responsible for generating the memorizable Nanog-low cell state that is prone to differentiate into PrE.

We thank this referee for an accurate description of our work and the recognition of its relevance.

Even though I am not an expert in Nanog regulation or ES cell biology, it was easy for me to appreciate that this is a very thorough study attempting to obtain some important insights on the mechanisms of stem cell fate decisions. Perhaps due to my distance to the field, I could verify that readers not familiar with the tools and techniques should be able to read this manuscript and distill the key insights with little background other than what is presented in the introduction.

© 2022. Published by The Company of Biologists under the terms of the Creative Commons Attribution License (https://creativecommons.org/licenses/by/4.0/).

We appreciate that this referee has found the study intelligible and thorough in our goal of providing a better mechanistic understanding of stem cell fate decisions.

The figures and supplementary data are well-presented and concise enough to understand the key experimental evidence that supports the conclusions. The methods are also described in detail, enough to allow anyone with minimal ES cell culture knowledge and basic molecular biology training to reproduce the large majority of results. **Perhaps the statistical analyses are a bit more obscure and hard to interpret from looking only at the figures or figure legends.** Only a few of these data tables are present and in some cases, **the number of experiments performed is not easy to obtain**. In addition, it is unclear if any statistical approach was followed to formally challenge some of the most important hypotheses. As an example, **in Fig. 2C, the** Nanog levels of WT and K9-KO cells are compared using a histogram plot, and it is claimed that Nanog heterogeneity is lost in K9-KO cells compared to WT. At least a simple statistical comparison of variation measures (i.e. compare the variance across n=5 independent culture+staining experiments) should be included. Although I cannot be thorough here, I have noted that this type of issue showed up on more than one occasion: i.e., authors stating a conclusion but with statistical analysis unclear or not shown (I also couldn't find **any details on statistical methods in the Supplementary Methods**). While this is the most important major concern for me, I do think that it is easy to fix, and should take authors a reasonable amount of time to take care of it.

We have statistically tested all the results presented in the manuscript; we hope sufficient details are given in the legends of the figures. For the more specific comment of this referee regarding NANOG heterogeneity, we understand its rational. However, the meaning we and the stem cell community gives to "heterogeneity", particularly of NANOG, is not well captured by standard measurements such as the variance or the coefficient of variation. These measurements are useful to compare the dispersion of protein expression values across a population where all cells express the protein under study. In the case of NANOG, what is generally understood by heterogeneity is the presence of cells lacking NANOG expression or characterised by particularly low levels, regardless of the spread of values within the NANOG-positive/high compartment. This has been clarified on page 3: "*ES cells also exhibit extensive Nanog heterogeneity, characterised by a subpopulation expressing no or extremely low levels of NANOG.*". This is important because the variance or coefficient of variation between two populations can be extremely similar but since all expression values are higher in one condition, the NANOG-low compartment is mechanically depleted: this is basically what we oberve in our mutants as was already precisely described (page 6 of the new version: "*a clear shift in NANOG expression, leading to a strong reduction of the proportion of cells expressing no or low NANOG*").

As a more challenging aspect that would improve the manuscript, I believe deleting the K9me region in Nanog-GFP cell lines would help validate the heterogeneity conclusions drawn just from IF studies.

The experiment is indeed of interest. However, although important, we believe that this is not the main focus of the paper, particularly of this new version where we provide further molecular details of the link between H3K9me3, heterogeneity and primitive endoderm differentiation. More specifically, we have now analysed two additional mutants (Triple Negative KO cells for *Dnmt1/3a/3b* - TKO - and *Zfp57* KO - ZKO - cells) where H3K9me3 at *Nanog* is largely depleted (Fig.R1). In both mutants, NANOG heterogeneity is strongly compromised, as shown in Fig.R1E. This data is presented in Fig.6. We hope this will allow the referee to further agree with the conclusion that the lack of H3K9me3 at *Nanog* is associated with a less prominent proportion of cells expressing low or no NANOG. Furthermore, to address the criticism that our conclusion was "drawn just from IF studies", which was correct, we have also performed smFISH of *Nanog* mRNA to have an independent measure of heterogeneity in our mutants and hopefuly convince this referee of the validity of our conclusions. This is detailed below, in a related comment (minor point 4).

© 2022. Published by The Company of Biologists under the terms of the Creative Commons Attribution License (https://creativecommons.org/licenses/by/4.0/).

My other, more "minor" concerns are presented in bullet-point style:

1) Without 2i, Nanog+ cells seem to have almost normal levels of H3K9me in the intermediate region element that is identified in this study (about 2% of IP, based on the y-axis units). Sure, in comparison, Nanog- cells have much higher levels of H3K9me, but Nanog+ cells seem to have quite a bit of K9me still... I'm not sure whether something so arbitrary as "% of IP material" can be assessed in such absolute terms as I'm doing here. But, what do authors make of this result, regarding their conclusions about Nanog epigenetic memory and timing of differentiation... **Is it possible that K9me begins to be deposited in Nanog+ cells, but then the timing involves further deposition of K27me3?**

The referee is perfectly right when comparing our datasets: it is true that *Nanog*-GFP+ve cells can present H3K9me3 enrichment at *Nanog*. In the original manuscript we did not elaborate on this observation because the *Nanog*-GFP reporter has some limitations regarding the half-life of the GFP, much longer than that of NANOG: it was possible that a fraction of *Nanog*-GFP+ve cells had no active NANOG expression and these could be the cells detected by H3K9me3 ChIP. However, we show now that when the same cells are selected with Puromycin, which is linked to the GFP allele and therefore leads to a pure population of NANOG-expressing cells, H3K9me3 is still detected (see Fig.R2 in our responses to Reviewer 1). This indicates that H3K9me3 can be enriched at *Nanog* before *Nanog* downregulation, exactly as suggested by this referee.
The question then, as rightly asked by this referee, is how the transition to irreversible silencing is mediated. Part of the answer is difficult to address, as it involves losing the dependency to ERK. This may be directly or indirectly connected to recent findings by the Hackett lab (Reviewer 3), showing that epigenetic repressive inheritance is established during differentiation (PMID:35199868), as our work also illustrates. The other part of the answer may involve additional events occurring at the locus. What our data suggests is that it is the spreading of H3K9me3 to the *Nanog* promoter that is linked to the more robust silencing of *Nanog*. This was already obvious in *Nanog*-GFP-ve cells (Fig.1D of the current version), as well as in cells having terminally silenced *Nanog* (e.g. MEFs in Fig.3A), as shown in the original ms. We now show that this spreading, takes place after d3 during 2iOFF differentiation (Fig.R5, see the inset zooming around the *Nanog* TSS), when *Nanog* is irreversibly silenced. In mutant cells, however, H3K9me3 does not accumulate at any analysed time point (Fig.R5). This data has been added as Fig.3B and Fig.S3C and is described on page 7: "*We observed a step-wise increase of H3K9me3 (Fig.3B): if it remained low during the first 48h, it suddenly appeared after 3 days and increased at days 4 and 7, when low but clear signs of spreading to the promoter were also observed. In ΔK9 clones, however, H3K9me3 remained absent during differentiation (Fig.S3C).*" and "*while Nanog expression continued to decrease during differentiation of wild-type cells, when H3K9me3 further increased and then spread to the Nanog promoter (Fig.3B), ΔK9 cells displayed a stabilisation of low Nanog expression after the sharp decrease occurring during the first 2 days (Fig.3C)*". These analyses address more clearly how *Nanog* is effectively locked in the negative state during differentiation and we feel explanations calling H3K27me3 into play, although interesting, are beyond the immediate scope of our ms.

© 2022. Published by The Company of Biologists under the terms of the Creative Commons Attribution License (https://creativecommons.org/licenses/by/4.0/).

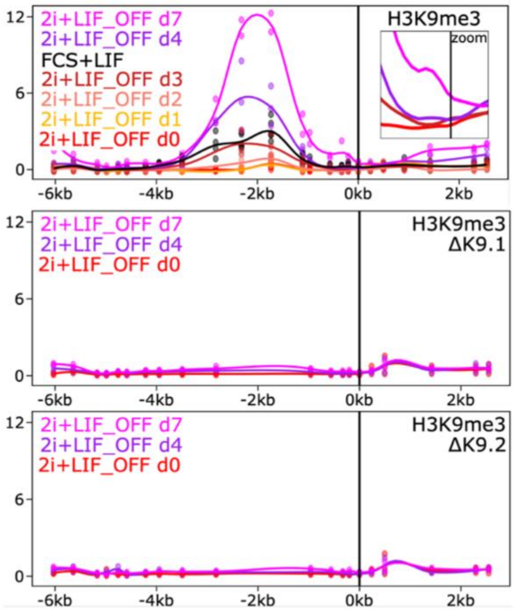

Fig.R5: Detailed analysis of H3K9me3 dynamics during ES cell differentiation in wt (top) and ΔK9 mutant cells (middle and bottom).

2) Deletion of the IR element (ΔK9) leads to higher steady-state Nanog expression. Is the difference between WT and ΔK9 cells lost in the presence of the ERK inhibitor?

3) How necessary is this element for ERK-related effects on ES cells? For example, do ΔK9 cells with Chir+LIF resemble 2i+LIF WT cells?

We will address points 2 and 3 together since they are closely related. The referee's point is interesting, asking if either for *Nanog* (point 2) or more generally for ES cells (point 3) the effects of ERK can be solely explained by H3K9me3 enrichment at *Nanog*. We now show that: (i) in the absence of the region enriched for H3K9me3, NANOG expression is still higher than that measured for wild-type cells when the cells are cultured in 2i+LIF (Fig.R6A); (ii) *Nanog* expression is upregulated when ΔK9 cells are treated with the ERK inhibitor (Fig.R6B); (iii) the cells display typical morphological changes in 2i+LIF (Fig.R6C). ERK impacts ES cell biology through a plethora of mechanisms and we did not expect all the effects to be mediated neither by H3K9me3 at *Nanog* nor by *Nanog* regulation. This data is available in the new version in Fig.S1C,S3A,S4B,S6. A comment has been added on page 6: "*the loss of heterogeneity was not as prominent as the one achieved by ERK inhibition (Fig.S1D), indicating that ERK also inhibits Nanog transcription by other means. In line with this, Nanog expression further increased upon ERK inhibition in ΔK9 clones (Fig.S1C).*".

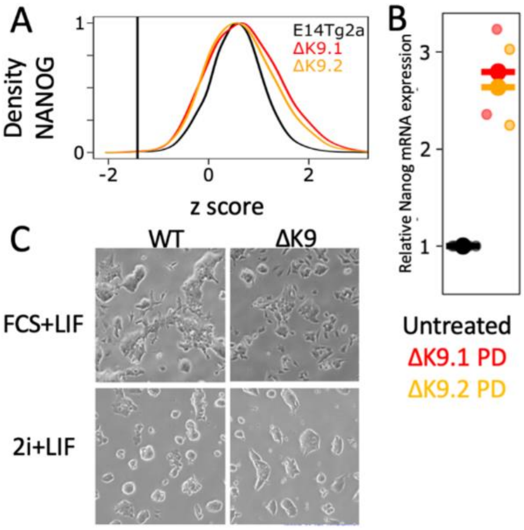

© 2022. Published by The Company of Biologists under the terms of the Creative Commons Attribution License (https://creativecommons.org/licenses/by/4.0/).

Fig.R6: Responses of ΔK9 cells to PD and 2i. **(A)** Expression of NANOG in wt and ΔK9 cells cultured in 2i+LIF. **(B)** Expression of Nanog upon PD treatment of ΔK9 cells. **(C)** Morphological changes of 2i cultures in wt and ΔK9 cells.

4) A major conclusion seems to be the loss of Nanog heterogeneity upon K9me element deletion. Do any other experiments support this "loss of heterogeneity"? Perhaps performing colony (or single-cell culture) assays and staining with Nanog IF would be helpful?

We are not sure of understanding the referee's comment and how colony or single-cell culture would help reinforcing the reduction of *Nanog* heterogeneity. To try to meet the referee's concern, however, we have performed smFISH of *Nanog* mRNA in wild-type and mutant cells (Fig.R7). In contrast to wild-type cells, where a substantial number express less than 5 *Nanog* mRNA molecules, ΔK9 cells exhibit less cells with no/low expression and an enrichment of cells expressing 11 to 50 molecules. This data has been added as Fig.S1E,F and is described on page 6: "*Nanog mRNA levels were slightly upregulated in ΔK9 cells (Fig.2B), which presented a clear shift in NANOG expression, leading to a strong reduction of the proportion of cells expressing no or low NANOG (Fig.2C), as confirmed at the mRNA level by smFISH (Fig.S1E,F)*.".

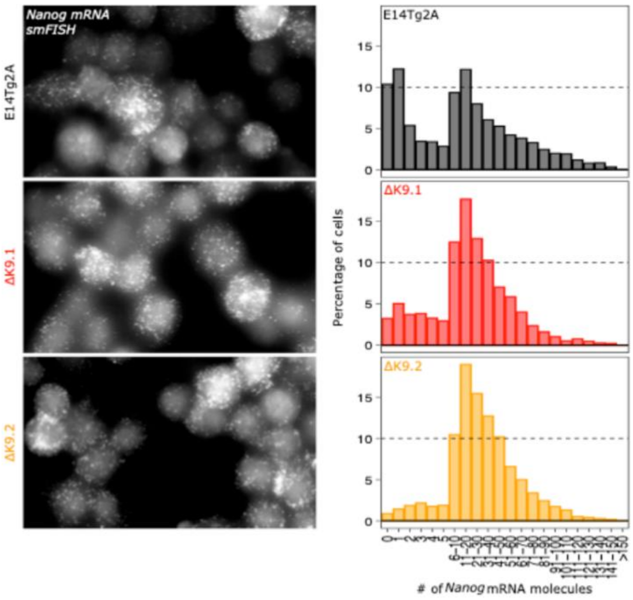

Fig.R7: Analysis of *Nanog* heterogeneity by smFISH.

5) In fig 2A, there seem to be much lower H3K9me levels across the entire Nanog gene body, and not just in the deleted región suggesting a deterministic role for this region (perhaps as an initiator of gene silencing)... What happens with LIF withdrawal? Does K9me appear gradually, even in spite of the deleted element? Is the silencing just based on K27me3 accumulation (independent of K9me)?

This point is directly connected to minor point 1 made by this referee. Indeed, the levels of H3K9me3 within *Nanog* itself are low but above those observed in our mutants. We believe, as the referee also suggests, that this has an important role in the effective silencing of *Nanog*. The new data presented in Fig.R5 directly addresses this by showing that there is indeed some level of spreading to the *Nanog* promoter during differentiation. In the same Fig.R5 we also address the question of whether H3K9me3 does encroach at the locus in our differentiating mutants: it is not the case, further showing that H3K9me3 is not involved in initially downregulating *Nanog* but in locking the silent state to facilitate the irreversible exit from pluripotency.

6) A big assumption of the conclusions is that K9me is the most important thing that happens to that region, but this is based only on the analysis of a panel of ChIP with anti-modified-histone antibodies... Does any transcription factor bind there?

© 2022. Published by The Company of Biologists under the terms of the Creative Commons Attribution License (https://creativecommons.org/licenses/by/4.0/).

We thank the referee for this question, which has prompted us to perform a detailed analysis of the region using online ressources such as cistrome (http://dbtoolkit.cistrome.org/). This software enables to interrogate which factors have been identified by ChIP-seq to bind any desired genomic interval. When we analysed the deleted region a major TF appeared as a strong positive hit: ZFP57, a zinc finger TF known to bind the TGC**CG**C consensus when it is CpG methylated to trigger H3K9me3. Satisfactorily, other proteins involved in H3K9me3 were also recovered, such as KAP1 and DNA methyltransferases (Dnmt3a). Two other TFs could be identified: SMAD2/3, which have been shown to bind H3K9me3 to initiate activation processes (PMID:22196728) and may provide interesting insights for future projects addressing how BMP signalling may deplete the region of H3K9me3 and hence oppose ERK activity; PRDM15 (belonging to a family with extensive links to histone methylation, PMID:33774927). The hits obtained with cistrome are presented in Fig.S7A. Among the identified factors, ZFP57 was particularly appealing because 2 DNA motifs could be identified close to the H3K9me3 peak, the region is CpG- methylated in FCS+LIF but not in 2i+LIF (Fig.R1A,B), and we could validate its binding by ChIP-qPCR in wild-type cells grown in FCSLIF but neither in 2i+LIF nor in *Dnmt1/3a/3b* TKO cells (Fig.R1C). These observations were the trigger of a new whole set of analyses of ZFP57, as described in Fig.R1 and on pages 10/11 of the new ms.

7) How do the authors propose that ERK actually regulates this epigenetic mechanism? Additional evidence would be helpful here. For instance, how fast can ERK inhibition cause loss of H3K9me?

Based on our new results on ZFP57, which is indeed essential to trigger H3K9me3 at *Nanog* as shown by analysing H3K9me3 in *Zfp57* KO (ZKO) and in *Dnmt1/3a/3b* TKO cells (Fig.R1), we believe that the effect of ERK on the locus is mediated by its indirect regulation of DNA methylation. In the absence of ERK activity, DNA methylation is abrogated and so is ZFP57 binding, leading to the loss of H3K9me3 (Fig.R1A-C). This may explain the kinetics of H3K9me3 loss upon ERK inhibition, which we had already show to take 2-3 days, a timing compatible with passive dilution of DNA methylation. This data is now available in Fig.6, described on pages 10/11 and discussed on page 11/12:

8) An aspect of Nanog epigenetic regulation by ERK still eludes my conscience: Say you take Nanog negative cells and positive cells and separate them by FACS. After separating, both populations are treated with the PD inhibitor. Then PD is washed out. Do previously Nanog-negative cells turn Nanog down faster than the positive ones? This might help clarify whether just ERK or other additional pathways might be contributing to the Nanog memory function.

We acknowledge the elegance of the experiment proposed by this referee. However, and as mentioned above, PD requires 2-3 days to deplete H3K9me3 from the region. Similarly, PD withdrawal may take several days to enable the restoration of H3K9me3. Therefore, although appealing, the experiment is not trivial. Moreover, it has now been well established that *Nanog*-negative cells are also partially eliminated from the cultures upon PD treatment (PMID:29779897). Therefore, this experiment has too many confounding factors to provide clear answers.

9) Plasticity of Nanog state: Authors state that Nanog cannot be turned on by PD after a certain differentiation switch has occurred, and they show that ERK inhibition does not lead to K9me changes in the Nanog intermediate regions within MEFs. But what about differentiating ES cells? Authors could try PD treatments 1-3 days after LIF removal (instead of putting it on MEFs) - At which point during differentiation is the decision to turn into XEN reversible?

As for the previous point, this is an appealing experiment that is unfortunately impossible to be made in such way that clear conclusions can be inferred, mainly due to the long time required to deplete H3K9me3 from *Nanog* upon PD treatment, which is not adequate to perform the assay as cells differentiate: to expect a depletion of H3K9me3 at around d4 we would need to add the inhibitor very early after triggering differentiation, which may itself be altered due to pleiotropic effects related to ERK inhibition, a major player in ES cell differentiation.

© 2022. Published by The Company of Biologists under the terms of the Creative Commons Attribution License (https://creativecommons.org/licenses/by/4.0/).

Reviewer #2 (Significance (Required)):

The advance presented is mostly incremental instead of paradigm-shifting. However, due to the importance of understanding Nanog regulation in pluripotency and the lack of mechanistic insights about its epigenetic regulation, this is a highly impactful study.

We thank this referee for acknowledging that, although incremental, our study will be highly impactful.

Previously published knowledge has focused more on gene regulatory networks, other chromatin modifications, or broad unbiased whole-transcriptome analyses. Instead, this study presents a more nuanced, focused, and precise dissection of the Nanog epigenetic regulatory elements. As such, from a biologist's perspective, it is interesting as it shows a very interesting model for how stem cell epigenetic memory might actually be regulated. Yet, from a medical perspective, it should still be an interesting read for those looking for ways to manipulate Nanog expression or other genes in ES cells exiting quiescence (either for therapy or engineering).

We really appreciate that this referee has found our study nuanced, focused and precise.

Referee Cross-commenting

I generally agree with the other 2 reviewers. **I think that the experiment with dCas9 fused to epigenetic regulators that both reviewers seem to suggest might be very interesting to perform, although I believe that it could be very challenging (and still imperfect in many other ways**). A bit **more bioinformatic analysis of the K9me region** and additional probing of its dynamics, plus some **additional angles of evidence of heterogeneity**, could be enough proof. Granted, if important regulatory TF motifs are in the deleted region, or, if there are other lines of evidence that the region might be involved in Nanog activation by other means, then this will require either toning down some important conclusions or performing more specific experiments (either performing smaller deletions or using dCas9-fused histone modifiers, indeed).

We hope that with the new data added all the concerns shared by the referees are addressed.

Reviewer #3 (Evidence, reproducibility and clarity (Required)):

In this study, Dubois et al investigate the underlying mechanisms that enable (naïve pluripotent) cells to dynamically transition between distinct gene expression 'states'. Further they ask how such regulated heterogeneity is linked with lineage diversification and fate-decisions during cellular commitment phases. Using dynamic Nanog regulation as a paradigm, the authors initially map chromatin changes associated with expression states, and subsequently focus on a genomic region that acquires H3K9me3 coincident with expression status and/or cell type, and which shows a degree of heritability. They go onto to genetically delete this putative 'epigenetic' control region and elegantly show through a sequential molecular and cellular assay that it is linked with Nanog dynamics/stable silencing and ultimately cell fate. This implicates H3K9me3 as an epigenetic mechanism underlying cellular heterogeneity, which in turn feeds into acquisition of lineage identity during differentiation, particularly primitive endoderm in this case.

We thank this referee for a precise description of our work.

This is an elegantly -designed and -performed study that carefully maps molecular events and mechanisms that have broad relevance for understanding development. Moreover, the study emphasises how multiple mechanisms integrate differentially to regulate gene expression programmes in distinct contexts (e.g. the switchable influence of ERK signaling on H3K9me3 in pluripotent vs differentiated cells). This feeds into general concepts of what constitutes 'epigenetic' control. The study is well-written and cohesive.

© 2022. Published by The Company of Biologists under the terms of the Creative Commons Attribution License (https://creativecommons.org/licenses/by/4.0/).

We acknowledge the referee to recognise the importance of our study. We particularly appreciate his comment on how our work will nurture conceptual understanding of epigenetics.

Nevertheless, I have some comments on **whether the clear readouts the authors observe can be attributed specifically to H3K9me3 based on the perturbations performed**. Given this is central to their conclusions, **further exploration of the direct functional importance of chromatin states per se (H3K9me3 or other) for Nanog heterogeneity/dynamics could be useful to strengthen conclusions.** Some comments and suggestions along these lines are made below. With modifications and clarifications, I support this manuscript for publication.

We thank the referee to support publication of our study, provided that we clearly address important comments he made. We believe that the new mutants that we exploit (*Dnmt1/3a/3b* TKO and *Zfp57* KO) will fully mitigate his concerns and, in fact, provide improved mechanistic details.

Major comments.

1.) Distinguishing between genetic and epigenetic effects.
The authors identify a region of the upstream Nanog promoter based on its strong enrichment of H3K9me3 specifically when Nanog is heterogenous and/or silenced. They hypothesized this acquired H3K9me3 could be important for expression dynamics and indeed it correlates well. To functionally test this the specific region is deleted with CRISPR and these cells are referred to as Delta-"K9". A concern here is that whilst indeed the region of H3K9me3 has been removed, so has the underlying DNA sequence. **In principle, the effects observed downstream (Nanog expression, cell fate skewing etc) could reflect the absence of key TF, repressor, or insulator motifs within this region, or perturbation to genomic contact sites, rather than absence of H3K9me3 per se.** I think this needs at least further clarification/discussion in the text since the manuscript refers solely to these cells as lacking H3K9me3 at the assayed region. The discrepancy should ideally be tested experimentally to draw sturdy conclusions. For example, by using existing dCas9-based epigenetic editing tools to **perturb chromatin states without genetic manipulation**. Here, use of a KRAB (in Nanog positive) or VPR (in Nanog negative cells) could provide some information on a causal effect of depositing/perturbing H3K9me3 states directly (well upstream of Nanog TSS). Even better would be to specifically deposit of remove H3K9me3 using a programmable (de)methylase. Taking a less precise but more tractable approach could involve siRNA of the relevant H3K9me3 methylase and/or inhibitors to link Nanog activity with global H3K9me perturbation. More generally, **more information on the function/attributes of the deleted segment would be valuable**. What TF motifs are there? What loci does it loop to in contact maps? Is it CpG-poor/rich? What features seem to underlie its H3K9me3 targeting? etc. Indeed, it could be interesting in future to make more precise genetic perturbation of the locus (inversion, specific motif deletion etc) to dissect its key control function.

We fully agree with the referee's point concerning the difficulties to disentangle the role of H3K9me3 from other unknown properties provided by the DNA sequence itself, a point that was also raised by the other referees. Since we now show that cells carrying a fully wild-type *Nanog* locus but defective H3K9me3 (*Dnmt1/3a/3b* TKO and *Zfp57* KO cells) completely phenocopy our observations made in ΔK9 cells (Fig.R1), we hope that this criticism will be considered as fully addressed. Indeed, we show that these cells display reduced NANOG heterogeneity and impaired in PrE differentiation. All this new data, which we believe largely rules out additional effects of the DNA sequence in our reported observations, are now included as Fig.6, described on page 10/11 and discussed on pages 11/12.

The referee also requests more details about the nature of the deleted region, particularly on TF DNA binding motifs, as well as more general information about a multitude of aspects such as 3D topology. Concerning the former, we hope that our reply to Reviewer 2 minor point 6, with all the new information on ZFP57, will answer his question. For the later, we believe that describing other general features such as 3D contacts is out of the scope of this manuscript.

2.) Function of other chromatin marks at the regulatory region. The authors focus on H3K9me3 since it shows the greatest discrepancy between 2i (active) and Serum/Lif (heterogenous) ESC, and correlates well with read-outs. Nevertheless, H3K4me2 and histone

© 2022. Published by The Company of Biologists under the terms of the Creative Commons Attribution License (https://creativecommons.org/licenses/by/4.0/).

acetylation at the same genomic locus also shows this correlation (inversely) (Fig 1B). It could be that changes in these chromatin states are upstream and/or causal of the observed changes in H3K9me3. **In other words can the authors comment on the possibility that Nanog activity is linked with dynamic deposition of activating marks such as acetylation, which may or may not in turn be linked with the observed H3K9me3 dynamics**.

We thank the referee for this comment and we acknowledge that additional information on the chromatin status of the locus could be, in principle, of interest. We do indeed observe opposite variations for some active histone marks, as mentioned by this referee, most notably H3K4me2 (Fig.R8). In fact, we have extensive data on a related project describing how the whole locus becomes transcriptionally active in 2i+LIF, with RNAPol2 transcription starting from the -5kb enhancer and going up to the *Nanog* promoter (and also increased antisense transcription increasing from the *Nanog* promoter towards the -5kb enhancer) and several active histone marks being overenriched throughout the whole locus (Fig.R8). Whether this is affecting how ZFP57/H3K9me3 is recruited to the locus is, we believe, interesting but beyond the immediate current scope of our manuscript. We would prefer to leave all this information for a future potential manuscript; however, should this Reviewer demand its inclusion, we will do so.

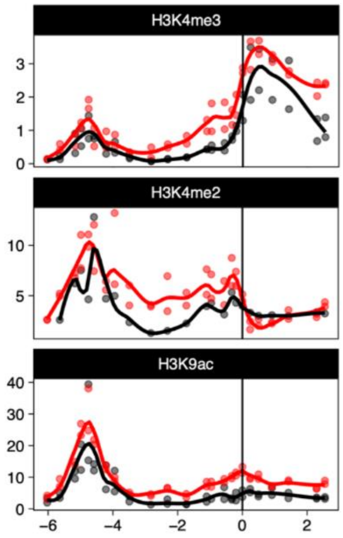

Fig.R8: Analysis of euchromatic histone marks at the *Nanog* locus.

3.) Function of H3K9me3. Whilst the authors convincingly demonstrate that when the H3K9me3 harboring region is deleted it leads to a number of important molecular and cellular consequences (Nanog expression dynamics, and cell identity/fate), it is unclear how H3K9me3 deposition >1kb from the TSS might exert such function. Indeed, in FCS+LIF ESC **or during differentiation H3K9me3 still does not encroach near the TSS.** It would be interesting to discuss or test the potential molecular mechanisms by which distal H3K9me3 functions, given the important influence it seems to have on locking down Nanog silencing. Does it affect chromatin compaction, contacts, TF binding?

This is an important comment. However, we showed already that in somatic cells, as well as in *Nanog*- GFP-ve cells, H3K9me3 does spread to the *Nanog* promoter (see Figs. 1D and 3A; see page 5: "*We observed that H3K9me3 was more prominent in Nanog-negative cells, with clear spreading towards the promoter (Fig.1D)*" and page 7: "*Therefore, we conclude that while H3K9me3 is found at Nanog in the three cell types analysed, its absolute levels and the degree of spreading towards the promoter are variable*". Despite this clarification, the referee particularly points to a lack of H3K9me3 encroachment at the *Nanog* promoter during ES cell differentiation, which is a very valid observation from the data that was previously available. To address this, we have analysed H3K9me3 at later differentiation time- points and we do observe H3K9me3 at the *Nanog* promoter region from d4 to d7 of differentiation, when cell fate decisions become irreversible. The level of spreading over the *Nanog* promoter attained during differentiation is similar to that observed in some non-pluripotent cells, such as XEN or TS cells.

© 2022. Published by The Company of Biologists under the terms of the Creative Commons Attribution License (https://creativecommons.org/licenses/by/4.0/).

This data is available in Fig.R5 (minor point 1 of Reviewer 2) and has been included in Fig.3B and commented on page 7: "*We observed a step-wise increase of H3K9me3 (Fig.3B): if it remained low during the first 48h, it suddenly appeared after 3 days and increased at days 4 and 7, **when low but clear signs of spreading to the promoter were also observed**. In ΔK9 clones, however, H3K9me3 remained absent during differentiation (Fig.S3C). Somehow unexpectedly, the appearance of H3K9me3 at d3 did not correlate with a particularly strong reduction of Nanog expression (Fig.3C). In fact, we observed Nanog downregulation taking largely place during the first 48h, in the absence of high levels of H3K9me3. **However, while Nanog expression continued to decrease during differentiation of wild- type cells, when H3K9me3 further increased and then spread to the Nanog promoter** (Fig.3B), ΔK9 cells displayed a stabilisation of low Nanog expression after the sharp decrease occurring during the first 2 days (Fig.3C)*".

4.) Statistics. There **is a general absence of statistics applie**d. For clarity, it would be useful to add appropriate statistical tests to quantitative measurements throughout (e.g. Fig 1A, 2B, 2D, 3C, 3D, 4A, 4D). All statitistical assessments are now implemented and described in the legends of the figures.

Jamie Hackett; assignment accepted: 26 July 2021; completed: 05 Aug 2020. My standard policy is to sign and date ALL peer review reports, irrespective of my comments or recommendations. Further communication related to this should be via the editorial office. Please do not remove this note.

Reviewer #3 (Significance (Required)):
The study will provide a valuable insight to the community and provide an intriguing cellular example of how and when gene regulation becomes 'epigenetic' - independent of inducing signals and reliant on pre-existing state. This feeds into understanding how cell fates become allocated and how cells acquire competence to respond to different cues during development.

We thank this referee for acknowledging the importance of our work regarding current conceptual discussions on the nature of epigenetic regulation, which are of primarily importance to understand developmental progression.

Original submission

First decision letter

MS ID#: DEVELOP/2022/201074

MS TITLE: H3K9 tri-methylation at Nanog times differentiation commitment and enables the acquisition of primitive endoderm fate

AUTHORS: Agnes Dubois, Loris Vincenti, Almira Chervova, Maxim V.C. Greenberg, Sandrine Vandormael-Pournin, Deborah Bourc'his, Michel Cohen-Tannoudji, and Pablo Navarro

I have now received all the referees reports on the above manuscript, and have reached a decision. The referees' comments are appended below, or you can access them online: please go to BenchPress and click on the 'Manuscripts with Decisions' queue in the Author Area.

The overall evaluation is positive and we would like to publish a revised manuscript in Development. Referee 2 suggests that the Abstract needs to be worded more carefully in order to accurately represent the results, I would encourage you to follow these suggestions. If you do not agree with any of their criticisms or suggestions explain clearly why this is so. If it would be helpful, you are welcome to contact us to discuss your revision in greater detail. Please send us a point-by-point response indicating your plans for addressing the referee’s comments, and we will look over this and provide further guidance.

© 2022. Published by The Company of Biologists under the terms of the Creative Commons Attribution License (https://creativecommons.org/licenses/by/4.0/).

Reviewer 1

Advance summary and potential significance to field

As one of the original reviewers, I reaffirm that I believe this manuscript makes a substantial contribution to the fields of stem cell biology, epigenetics, and differentiation. The manuscript provides novel mechanistic studies of Nanog epigenetic regulation by ERK, exploring reversible versus irreversible epigenetic control of gene expression and fate commitment. These should be highly significant and potentially of wide interest beyond just the readership of Development.

Comments for the author

In this revised version of the manuscript, Dubois et al. have successfully addressed the most important aspects of my previous criticisms, namely additional statistical reporting, additional evidence of reduced Nanog heterogeneity, and additional mechanistic insights on how ERK regulates Nanog heterogeneity through the identified K9me3 region. The authors have gone above and beyond to answer these comments and those of other reviewers, and they now even show that a specific DNAme-sensitive TF, ZFP57, binds to the K9 region and is responsible for Nanog heterogeneity. Furthermore, while new results provided show that ERK regulates Nanog levels in other ways, the authors identify that regulation of DNA methylation must be a major pathway controlling Nanog heterogeneity, which is partly explained by the binding of ZFP57. In sum, I consider my comments addressed and I am happy to recommend this manuscript for publication in its present form.

Reviewer 2

Advance summary and potential significance to field

Review of revised work - previous comments on significance still apply.

"The study is timely and interesting as it touches a number of topics: stable inheritance of chromatin modifications, link between signalling, chromatin status and transcription, how these influence differentiation, and heterogeneity. Therefore, this report would be of interest to a wide community."

Comments for the author

Thanks to the authors for engaging in constructive revisions. The new data provided certainly clears many of the doubts and addresses the concerns raised in all reviews, as well as providing an interesting link between previously disconnected observations.

The only remaining concert is about the wording of the abstract and the implications of causation which I suggest rephrasing. For example:

“We found that the transcription factor ZFP57, which binds methylated DNA to nucleate heterochromatin TRIGGERS histone H3 lysine 9 tri-methylation (H3K9me3) at Nanog.” [no data that shows Zfp57 triggers H3K9me3 deposition, only that ZKOs cells show loss of H3K9me3. The effects could be secondary to the genome-wide absence of Zfp57 binding (over multiple generations)].

“UPON THE LOSS of H3K9me3 at Nanog, ES cells display reduced heterogeneity of NANOG expression delayed commitment into differentiation and impaired ability to acquire a primitive endoderm fate.” [since neither stable deletion of Zfp57 or Dnmts is likely to cause only loss of H3K9me3 at the Nanog locus, this statement should be rephrased to more accurately describe the data.]

© 2022. Published by The Company of Biologists under the terms of the Creative Commons Attribution License (https://creativecommons.org/licenses/by/4.0/).

First revision

Author response to reviewers' comments

We thank our referees for a fair, fast and productive review. We agree with the comments made by one reviewer requesting to tone down some sentences in the abstract. We hope that the new version will be positively evaluated.

Thank you all for your help improving this manuscript.

Pablo Navarro

Second decision letter

MS ID#: DEVELOP/2022/201074

MS TITLE: H3K9 tri-methylation at Nanog times differentiation commitment and enables the acquisition of primitive endoderm fate

AUTHORS: Agnes Dubois, Loris Vincenti, Almira Chervova, Maxim V.C. Greenberg, Sandrine Vandormael-Pournin, Deborah Bourc'his, Michel Cohen-Tannoudji, and Pablo Navarro
ARTICLE TYPE: Research Article

I am happy to tell you that your manuscript has been accepted for publication in Development, pending our standard ethics checks.

© 2022. Published by The Company of Biologists under the terms of the Creative Commons Attribution License (https://creativecommons.org/licenses/by/4.0/).
